# ILC precursors differentiate into metabolically distinct ILC1-like cells during *Mycobacterium tuberculosis* infection

**DOI:** 10.1016/j.celrep.2022.110715

**Published:** 2022-04-19

**Authors:** Dan Corral, Alison Charton, Maria Z. Krauss, Eve Blanquart, Florence Levillain, Emma Lefrançais, Tamara Sneperger, Zoï Vahlas, Jean-Philippe Girard, Gérard Eberl, Yannick Poquet, Jean-Charles Guéry, Rafael J. Argüello, Yasmine Belkaid, Katrin D. Mayer-Barber, Matthew R. Hepworth, Olivier Neyrolles, Denis Hudrisier

**Affiliations:** 1 Institut de Pharmacologie et Biologie Structurale, IPBS, Université de Toulouse, CNRS, UPS, Toulouse, France; 2 Metaorganism Immunity Section, Laboratory of Host Immunity and Microbiome, National Institute of Allergy and Infectious Diseases, National Institutes of Health, Bethesda, MD, USA; 3 Lydia Becker Institute of Immunology and Inflammation, Division of Infection, Immunity, and Respiratory Medicine, School of Biological Sciences, Faculty of Biology, Medicine, and Health, Manchester Academic Health Science Centre, University of Manchester, Manchester M13 9PL, UK; 4 Institut Toulousain des Maladies Infectieuses et Inflammatoires (INFINITY), Université de Toulouse, CNRS, UPS, Toulouse, France; 5 Institut Pasteur, Microenvironment & Immunity Unit, 75724 Paris, France; 6 INSERM U1224, 75724 Paris, France; 7 Aix Marseille University, CNRS, INSERM, CIML, Centre d’Immunologie de Marseille-Luminy, Marseille, France; 8 Inflammation and Innate Immunity Unit, Laboratory of Clinical Immunology and Microbiology, National Institute of Allergy and Infectious Diseases, National Institutes of Health, Bethesda, MD, USA

**Keywords:** innate lymphoid cell, tuberculosis, mucosal immunity, immunometabolism

## Abstract

Tissue-resident innate lymphoid cells (ILCs) regulate tissue homeostasis, protect against pathogens at mucosal surfaces, and are key players at the interface of innate and adaptive immunity. How ILCs adapt their phenotype and function to environmental cues within tissues remains to be fully understood. Here, we show that *Mycobacterium tuberculosis* (*Mtb*) infection alters the phenotype and function of lung IL-18Rα^+^ ILC toward a protective interferon-γ-producing ILC1-like population. This differentiation is controlled by type 1 cytokines and is associated with a glycolytic program. Moreover, a BCG-driven type I milieu enhances the early generation of ILC1-like cells during secondary challenge with *Mtb*. Collectively, our data reveal how tissue-resident ILCs adapt to type 1 inflammation toward a pathogen-tailored immune response.

## Highlights

• Type 1 inflammation shapes the fate of lung IL-18Rα^+^ ILC
• IL-18Rα^+^ ILC give rise to protective ILC1-like cells during *Mtb* infection
• ILC1-like cells depend on glycolysis for their differentiation and function
• BCG vaccination promotes the early generation of ILC1-like cells in the lung

## Graphical abstract

**Figure:**
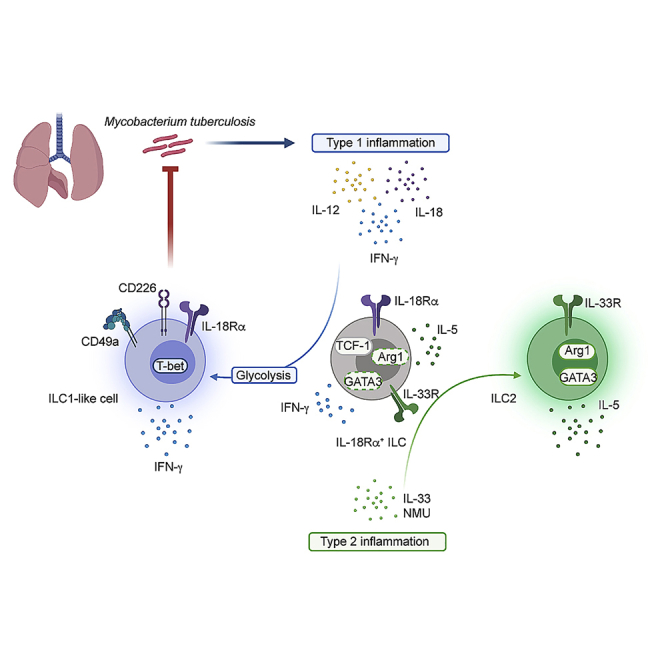

## Teaser

In this study, Corral et al. demonstrate that the inflammatory and metabolic environments shape the fate of lung ILC precursors expressing IL-18Rα. During *Mycobacterium tuberculosis* infection, lung ILC precursors differentiate into IFN-γ, producing ILC1-like cells. ILC1-like cells display the protective capacity to control the mycobacterial infection.

## Introduction

Innate lymphoid cells (ILCs) are a population of tissue-resident cells that play a key part in tissue homeostasis and immunity (Vivier et al., 2018). ILCs are subdivided into three distinct populations based on their expression of cytokines and specific transcription factors. ILC1 depends on T-bet and produce interferon-γ (IFN-γ), ILC2 depends on GATA3 and produces interleukin-5 (IL-5) and IL-13, and ILC3 expresses RORγt and produces IL-17A and IL-22 (Meininger et al., 2020; Vivier et al., 2018). Based on these properties, group 1, 2, and 3 ILCs contribute to type 1, 2, and 3 immune responses, respectively.

The regulome of ILCs evolves progressively during the development of each population to reach a state in which key loci specific to each lineage are acquired (Shih et al., 2016; Vivier et al., 2016). However, several elements controlling cytokine expression or loci encoding lineage-determining transcription factors remain broadly accessible in all ILC subsets (Shih et al., 2016). This feature contributes to the remarkable ability of ILCs to dynamically adapt to physiological or pathological alterations in their tissue of residence. Besides the local plasticity among mature ILC subsets (see Bal et al., 2020 and references therein), circulatory and tissue resident ILC precursors in humans and mice contribute to the local differentiation into mature ILCs, or *in situ* “*ILCpoiesis*” (Lim and Di Santo, 2019), sustaining the ILC response depending on tissue and inflammation (Ghaedi et al., 2020; Lim et al., 2017; Zeis et al., 2020). While the various populations of tissue-resident ILCs can promptly sense and adapt to environmental changes (Meininger et al., 2020; Ricardo-Gonzalez et al., 2018), the mechanism allowing such responses remains to be fully elucidated.

In both mice and humans, *Mycobacterium tuberculosis* (*Mtb*) infection induces prolonged proinflammatory responses that are associated with oxidative stress. *Mtb* infection is associated with metabolic changes in the lungs, involving the utilization of aerobic glycolysis primarily instead of oxidative phosphorylation (OXPHOS) in mitochondria (Warburg effect) (Fernández-García et al., 2020; Shi et al., 2015). The infection also leads to the development of type 1 immunity, which is mediated by IFN-γ and is associated with protection (O’Garra et al., 2013) in place of a type 2 environment at steady state.

Here, using the murine model of *Mtb* infection, we explored how lung ILCs respond and adapt to type 1 inflammation driven by chronic pulmonary infection. Our work uncovers the local differentiation of lung IL-18Rα^+^ ILCs into a protective ILC1-like population through an IFN-γ/Stat-1-dependent and metabolic reprogramming during *Mtb* infection.

## Results

### Local differentiation of lung ILC precursors into ILC1-like cells during *Mtb* infection

**Figure 1:**
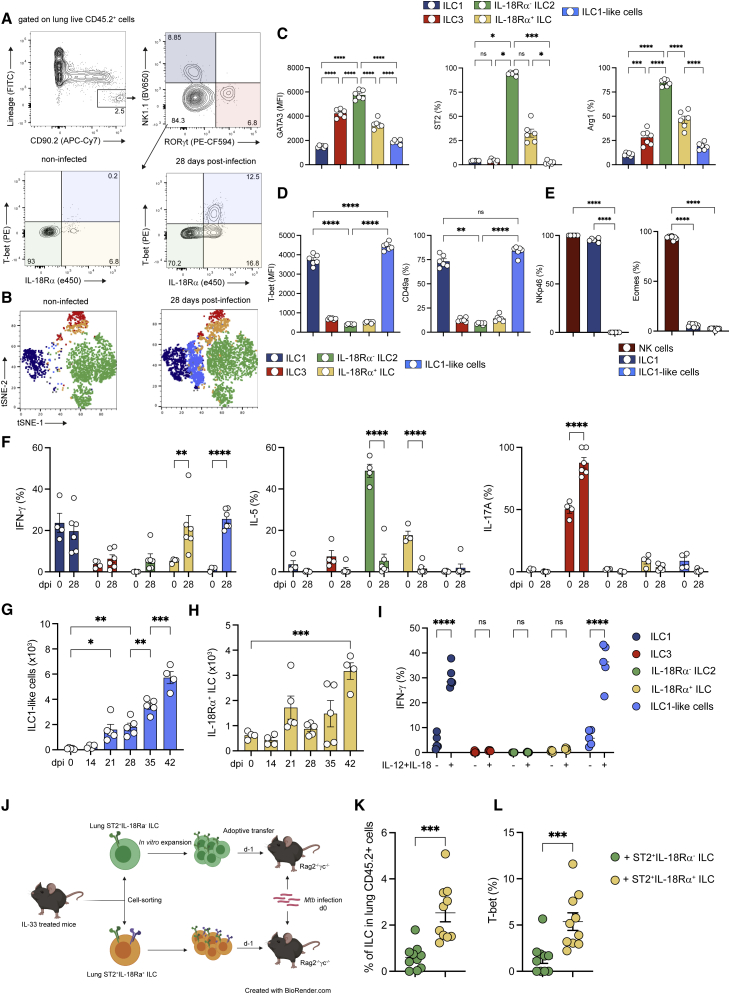
IL-18Rα-expressing ILC differentiate into ILC1-like cells during *Mtb* infection (A) Representative dot plots showing the gating strategy used for ILC subsets in the lungs of *Mtb*-infected C57BL/6 mice (top graphs): ILC1 (dark blue), ILC3 (red), IL-18Rα^−^ ILC2 (green), IL-18Rα^+^ ILC (yellow), and T-bet^+^IL-18Rα^+^ ILC (light blue). (B) Unsupervised t-distributed stochastic neighbor embedding (t-SNE) distribution of total lung Lin^−^CD90.2^+^ populations at steady state (left graph) and during *Mtb* infection (right graph). Based on the gating strategy defined in (A), ILC subsets were depicted with the same color code. (C) Expression of GATA3 (MFI), ST2 (%), and Arg1 (%) in indicated ILC subsets at day 28 post-infection in C57BL/6 mice. (D) As in (C), but for the expression of T-bet (MFI) and CD49a (%). (E) Percentages of NKp46^+^ (left) and Eomes^+^ (right) cells in ILC1-like cells compared to in NK cells and ILC1 at day 28 post-infection. (F) Percentages of IFN-γ, IL-5, and IL-17A-positive cells in the indicated ILC subsets after phorbol myristate acetate (PMA)/ionomycin stimulation at day 28 post-infection in C57BL/6 mice. (G and H) Absolute numbers of ILC1-like cells at the indicated days after *Mtb* infection (G) and absolute numbers of IL-18Rα^+^ ILC at the indicated days after *Mtb* infection (H). Before sacrifice, mice were injected with fluorescent anti-CD45.2 to distinguish vascular and parenchymal cells. ILC1-like cells and IL-18Rα^+^ ILC have been gated on lung-resident cells. (I) Percentages of IFN-γ^+^ cells in the indicated ILC subsets after *ex vivo* stimulation with IL-12+IL-18 or not at day 28 post-infection in C57BL/6 mice. (J) Experimental settings for the adoptive transfer of ST2^+^IL-18Rα^−^ and ST2^+^IL-18Rα^+^ ILC into Rag2^−/−^γc^−/−^ before *Mtb* infection. (K) Percentage of ILC (Lin^−^CD45.2^+^CD90.2^+^CD127^+^) in the lung at day 21 post-infection in Rag2^−/−^γc^−/−^ after adoptive transfer of ST2^+^IL-18Rα^−^ ILC (green) and ST2^+^IL-18Rα^+^ ILC (yellow). (L) As in (J), but for T-bet expression in ILCs. In (C), (D), (G) and (H), data are representative of 5 independent experiments; in (E), (F), and (I), data are representative of 2 independent experiments; and in (K) and (L), data are a pool of 2 independent experiments, with each symbol representing an individual mouse. Graphs depict data as means ±SEMs, and statistical analysis was performed using 2-way ANOVA (F and I), 1-way ANOVA (C–E and G–H) or the Mann-Whitney test (K and L). ^∗^p < 0.05; ^∗∗^p < 0.01; ^∗∗∗^p < 0.001; ^∗∗∗∗^p < 0.0001).

To investigate how a chronic type 1 infection affects lung ILC subsets, C57BL/6 mice were infected with the *Mtb* reference strain H37Rv. At steady state, *bona fide* lung ILCs were defined as a population that does not express lineage markers (CD3, CD4, CD8, T cell receptor-β[TCR-β], TCRγδ, CD49b, CD11b, CD11c, B220, CD19, F4/80, GR-1, TER119, and FcεR1a) but highly express CD90.2 (Figure 1A). ILC2 (GATA3^high^) was identified after the exclusion of ILC1 (NK [natural killer]1.1^+^) and ILC3 (RORγt^+^) cells (Figure 1A). At steady state, and in agreement with previously published work (Monticelli et al., 2011, Stehle et al., 2018; Vivier et al., 2018), the lung was dominantly enriched in ILC2 (Figures 1A and 1B). Notably, a small frequency of ILCs expressed IL-18Rα (Figure 1B), a phenotype previously used to identify tissue-resident ILC precursors (ILCPs) able to differentiate into ILC2 in the context of type 2 inflammatory responses (Ghaedi et al., 2020; Zeis et al., 2020). Based on our results, these cells are defined as Lin^−^CD90.2^+^CD45.2^+^NK1.1^−^RORγt^−^IL-18Rα^+^T-bet^−^ cells. IL-18Rα^+^ ILCs expressed GATA3 at lower levels than IL-18Rα^−^ ILC2 (Figure S1A). IL-18Rα^+^ ILCs formed a heterogeneous population biased toward an ILC2 profile given that 50% of the population expressed ST2 and Arg1 (Figure S1A). At the functional level, these cells produced lower amounts of IL-5 compared to IL-18Rα^−^ ILC2, and did not produce IFN-γ, as did ILC1 (Figure S1B). In line with previous studies (Ghaedi et al., 2020; Zeis et al., 2020), we found that almost all lung IL-18Rα^+^ ILCs expressed T cell factor-1 (TCF-1), like ILC2 precursors from the bone marrow, confirming their immature profile, regardless of ST2 or Arg1 expression (Figure S1C). *Mtb* infection had a profound impact on ILC composition and phenotype and was associated with the gradual increase in ILC1 and ILC3 (Figure S1D). Of particular interest, *Mtb* infection promoted the accumulation of an ILC population, not detectable at steady state, expressing both IL-18Rα and T-bet within the lung and that clustered closely with ILC1 and thus were called “ILC1-like cells” (Figures 1A and 1B), and the concomitant reduction in IL-18Rα^−^ ILC2 (Figure S1D). Phenotypical analysis revealed that this subset displayed little to no classical ILC2 markers, such as GATA3, ST2, Arg1, and IL-5 (Figure 1C), or ILC3 markers, such as RORγt (Figure 1A). Like *bona fide* ILC1, this subset expressed T-bet and CD49a (Figure 1D), but did not express NK1.1 (gated on NK1.1-negative ILCs), NKp46, or Eomes (Figures 1A and 1E). This population was able to produce IFN-γ, but not IL-5 or IL-17A (Figure 1F). ILC1-like cells became detectable after 21 days of infection and expanded in the following weeks (Figure 1G). *In vivo* antibody labeling with fluorescent anti-CD45.2 was performed before sacrifice during *Mtb* infection (Anderson et al., 2014), and ILC1-like cells were not labeled by injected antibodies, suggesting that they reside in the parenchymal lung tissue (Figure S1E). T cell infiltration in the lungs, which correlates with mycobacterial containment in immunocompetent animals, occurs at day 21 post-infection (Urdahl et al., 2011) and therefore coincides with the detection of ILC1-like cells. Thus, we assessed the role of adaptive immunity in the emergence of this subset. ILC1-like cells were detectable in *Mtb*-infected Rag2^−/−^ mice that lack T and B cells, and at a higher level than in infected wild-type mice (Figure S1F). Thus, the generation of ILC1-like cells following infection by *Mtb* does not require T or B cells.

ILCs have been reported to adapt their profile to environmental cues. Different mechanisms have been described to sustain the local adaptation of ILCs during inflammation such as plasticity of mature ILC subsets (Bal et al., 2020), *in situ* differentiation of ILCPs (Ghaedi et al., 2020; Zeis et al., 2020), and the migration of ILCs from the bone marrow (Zeis et al., 2020). Furthermore, ILCs with characteristics of ILC1-like cells have been shown to arise from various ILC subsets through mechanisms of plasticity, depending on the tissue and the inflammatory context (Bal et al., 2020; Gao et al., 2017; Klose et al., 2013; Silver et al., 2016; Vonarbourg et al., 2010). While ILC1-like cells do not express NK1.1 or NKp46 (Figures 1A and 1E), we sought to determine whether NK or ILC1 could differentiate into ILC1-like cells during *Mtb* infection. *In vivo* treatment of *Rag2*^*−/−*^ mice with anti-NK1.1 mAb during the infection fully depleted NK cells and ILC1 (Figure S1G), but only a modest reduction was observed for ILC1-like cells (Figure S1G). Intriguingly, the depletion of NK/ILC1 induced an accumulation of IL-18Rα^+^ ILC while not affecting IL-18Rα^−^ ILC2 (Figure S1G). These results suggest that either a small portion of ILC1-like cells could derive from NK/ILC1 or that one or more NK/ILC1-derived factors could contribute to the differentiation of ILC1-like cells during *Mtb* infection. In the lungs, ILC2 have been described to acquire the expression of T-bet, IL-18Rα, and IFN-γ during influenza virus infection (Silver et al., 2016). We hypothesized that ILC1-like cells could differentiate from lung ILC2. To assess whether ILC2 display plasticity during *Mtb* infection, we adoptively transferred total lung ST2^+^ ILC2, sorted regardless of their IL-18Rα expression, into *Rag2*^−/−^*γc*^−/−^ mice, which are devoid of T cells, B cells, and NK/ILCs, 1 day before *Mtb* infection (Figures S1H and 1I). To generate enough ILC2, we performed an *in situ* expansion of lung ILC2 by the intranasal administration of IL-33, leading to an induction of both ST2^+^IL-18Rα^−^ and ST2^+^IL-18Rα^+^ ILC (Figures S1H and S1I). Before transfer, we confirmed that sorted ST2^+^ILC expressed GATA3 but not T-bet or RORγt (Figure S1J) and noticed that IL-18Rα expression was lost during *in vitro* culture (Figure S1J). Following transfer, ILC2s upregulated T-bet in infected but not in non-infected mice (Figures S1K and S1L). Furthermore, T-bet^high^ daughter cells expressed higher levels of IL-18Rα compared to GATA3^high^ cells (Figure S1M). Given that lung ILC2s can give rise to ILC1-like cells, we sought to explore which ILC2 subset preferentially differentiated into ILC1-like cells. Intriguingly, while IL-18Rα^+^ ILCs displaying an ILC2 phenotype at steady state (Figure S1A) accumulate in the lungs during *Mtb* infection (Figure 1H), they gained the potential to produce IFN-γ and did not produce IL-5 (Figure 1F). However, IL-18Rα^+^ ILCs, unlike ILC1 and ILC1-like cells, did not respond to *ex vivo* stimulation with IL-12 and IL-18 to produce IFN-γ (Figure 1I). Thus, we hypothesized that this population could have the potential to differentiate into ILC1-like cells and could thus represent a precursor of ILC1-like cells. We assessed whether IL-18Rα^+^ ILC rather than IL-18Rα^−^ ILC2 has the potential to differentiate into ILC1-like during *Mtb* infection. To this end, we sorted ST2^+^ ILC2 subsets from IL-33-treated mice based on their IL-18Rα expression and adoptively transferred them into *Rag2*^−/−^*γc*^−/−^ mice 1 day before infection with *Mtb* (Figure 1J). Interestingly, at 21 days post-infection, we found that the transfer of ST2^+^IL-18Rα^+^ ILC resulted in higher proportions of ILCs in the lungs when compared to the conditions in which the same number of ST2^+^IL-18Rα^−^ ILCs were transferred (Figure 1K); in addition, T-bet expression among ST2^+^IL-18Rα^+^ ILCs was significantly increased (Figure 1L). Compared to the results obtained after the transfer of total ILC2 (Figure S1L), the reduced induction of T-bet expression after the transfer of ST2^+^IL-18Rα^+^ ILC (Figure 1L) suggests that ST2^+^IL-18Rα^−^ ILC could provide signals favoring the differentiation of ILC1-like cells. Thus, ST2^+^IL-18Rα^+^ ILCs, rather than ST2^+^IL-18Rα^−^ ILCs, have the potential to differentiate into ILC1-like cells during *Mtb* infection. IL-18Rα^−^ ILC2 cells represent the most mature population of ILC2, based on their expression of GATA3, Arg1, IL-5, and TCF-1 (Figures 1C, 1F, and S1A–S1C). Finally, to confirm that ILC1-like cells do not derive from mature, IL-5-producing ILC2, we crossed IL-5^Cre-tdTomato^ (Red5) mice with ROSA26^fl/stopYFP^ mice to enable fate mapping of mature ILC2 (Nussbaum et al., 2013). We observed that very few IL-18Rα^+^ ILCs and ILC1-like cells expressed IL-5 and yellow fluorescent protein (YFP) after *Mtb* infection, compared to IL-18Rα^−^ ILC2 (Figures S1N and S1O). These results demonstrate that mature ILC2 do not give rise to ILC1-like cells.

Taken together, our data show that *Mtb* infection differentially affects the composition of ILC subsets in the lung, and especially induces the local differentiation of lung ILC1-like cells from lung ILC2 precursors rather than from mature ILC2 or ILC1/NK cells.

### Type 1 inflammatory environment shapes the fate of IL-18Rα^+^ ILCs

**Figure 2:**
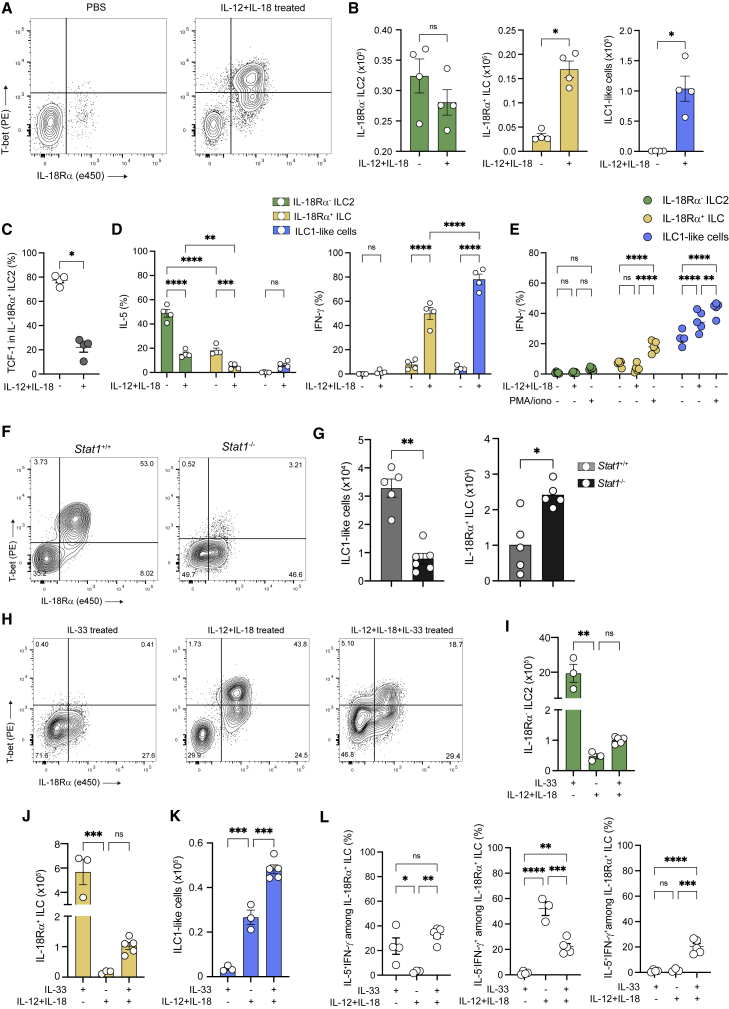
The inflammatory environment shapes the fate of IL-18Rα^+^ ILCs (A) Representative dot plots of T-bet and IL18Rα expression after intranasal administration of PBS and IL-12+IL-18 in *Rag2*^−/−^ mice in Lin^−^CD45.2^+^CD90.2^+^NK1.1^−^RORγt^−^ cells. (B) Absolute numbers of IL-18Rα^−^ ILC2 (green), IL-18Rα^+^ ILC (yellow), and ILC1-like cells (blue) after cytokine (IL-12+IL-18) or control (PBS) treatment. (C) Percentage of TCF-1 in lung IL-18Rα^−^ ILC2 after intranasal administration of PBS or IL-12+IL-18. (D) Percentages of cells expressing IL-5 or IFN-γ among the indicated ILC subsets after PMA/ionomycin stimulation. (E) Percentages of IFN-γ ^+^ cells in the indicated ILC subsets after *ex vivo* stimulation with IL-12+IL-18, PMA/ionomycin, or not from IL-12+IL-18-treated C57BL/6 mice. (F) Representative dot plot of T-bet and IL-18Rα expression in IL-12+IL-18-treated *Stat1*^+/+^*versus Stat1*^−/−^ mice. (G) Absolute numbers of ILC1-like cells (left) and IL-18Rα^+^ ILC (right) in *Stat1*^+/+^*versus Stat1*^−/−^ mice treated as in (F). (H) Representative dot plots of T-bet and IL18Rα expression after IL-33, IL-12+IL-18, and IL-12+IL-18+IL-33-treated *Rag2*^−/−^ mice in Lin^−^CD45.2^+^CD90.2^+^NK1.1^−^RORγt^−^ cells. (I–K) Absolute numbers of IL-18Rα^−^ ILC2 (I), IL-18Rα^+^ ILC (J), and ILC1-like cells (K) in IL-33, IL-12+IL-18, or IL-12+IL-18+IL-33-treated mice. (L) Percentage of IL-5^+^IFN-γ^−^ (right), IL-5^−^IFN-γ^+^ (center), and IL-5^+^IFN-γ^+^ (left) in IL-18Rα^+^ ILC in IL-33, IL-12+IL-18, and IL-12+IL-18+IL-33-treated mice. Each symbol represents an individual mouse. Statistical analysis was performed using Mann-Whitney (B, C, and G) and 1-way (I–L) and 2-way (D and E) ANOVA tests. ^∗^p < 0.05; ^∗∗^p < 0.01; ^∗∗∗^p < 0.001; ^∗∗∗∗^p < 0.0001. Graphs depict data as means ±SEMs. Data are representative of 3 (B, D, and I–K) and 2 (C, E, G, and L) independent experiments.

Next, we aimed to assess how the inflammatory milieu influences the fate of IL-18Rα^+^ ILC. *Mtb* infection triggers the development of a type 1 immunity (O’Garra et al., 2013). Both IL-12 and IL-18 contribute to the establishment of this type 1 inflammatory environment (Kinjo et al., 2002; O’Garra et al., 2013), in particular, by inducing the expression of IFN-γ on ILC1, NK cells, and T helper 1 (Th1) (Chiossone et al., 2018; Weizman et al., 2017). Therefore, we administered IL-12 and IL-18 to mice intranasally for 1 week and found that this treatment was sufficient to induce the accumulation of ILC1-like cells in the lungs (Figures 2A and 2B). Furthermore, IL-18Rα^+^ ILC also expanded in these settings (Figure 2B) and lost the expression of TCF-1 (Figure 2C), supporting the idea that these cells may undergo a local differentiation process. Similar to that during *Mtb* infection, IL-18Rα^+^ ILC and IL-18Rα^−^ ILC2 lost their ability to produce IL-5 and acquired the ability to produce IFN-γ following cytokine injection (Figure 2D). Interestingly, unlike ILC1-like cells, *ex vivo* stimulation with IL-12/IL-18 failed to induce IFN-γ in IL-18Rα+ (Figure 2E), suggesting that this subset does not fully acquire an ILC1 phenotype. Based on the expression of several markers (GATA3, Arg1, T-bet, IL-18Rα, CD49a, CD226, and Ki67), we found close similarities in both IL-18Rα^+^ ILC and ILC1-like cells generated upon either IL-12/IL-18 treatment or during *Mtb* infection (Figures S2A and S2B). Indeed, the administration of IL-12 and IL-18 to IL-5^Cre-tdTomato^ x ROSA26^fl/stopYFP^ mice confirmed that, in this model as well, ILC1-like cells do not derive from IL-5-producing mature ILC2 (Figure S3A), in agreement with results in Figure S1N and S1O.

Type 1 cues including IFN-γ are widely known to repress ILC2 function (Duerr et al., 2016; Moro et al., 2016) through STAT1 signaling. We therefore questioned the involvement of STAT1 signaling in the generation of ILC1-like cells. IL-12+IL-18 administration led to a 3-fold reduction in the differentiation of ILC1-like cells in *Stat1*^−/−^ mice, which cannot signal through type 1 and 2 IFNs as well as IL-27, compared to that in wild-type mice (Figures 2F and 2G). Interestingly, IL-18Rα^+^ ILC accumulated in *Stat1*^−/−^ mice after IL-12+IL-18 treatment (Figure 2G), suggesting that STAT1 is required for fully differentiating these cells into ILC1-like cells. IL-12 and IL-18 treatment repressed the ILC2 phenotype (GATA3, ST2) while inducing the expression of ILC1-like markers (CD49a, IFN-γ) on IL-18Rα^+^ ILC (Figures S3B and S3C). Importantly, this phenomenon was completely abrogated in *Stat1*^−/−^ mice. Furthermore, infection of *Stat1*^−/−^ mice with *Mtb* also resulted in the accumulation of IL-18Rα^+^ ILC and a 3-fold reduction in ILC1-like cell generation (Figure S3D). More important, a similar result was observed after *Mtb* infection of *IL-12p40*^*−/−*^ mice (Figure S3E), demonstrating a causal role for IL-12 in driving the emergence of ILC1-like cells during *Mtb* infection. Interestingly, an accumulation of IL-18Rα^+^ ILC associated with a reduction in ILC1-like cells was also observed in anti-NK1.1-treated mice (Figure S1G), suggesting that NK/ILC1-derived factors, such as IFN-γ, may be involved in establishing a type 1 inflammatory environment favoring an ILC1-like cell differentiation through T-bet induction in a STAT1-dependent manner. Confirming this hypothesis, blocking anti-IFN-γ antibody also inhibited ILC1-like cell differentiation in IL-12+IL-18-treated mice (Figure S3F). Altogether, these results demonstrate that the generation of ILC1-like cells observed during *Mtb* infection can be closely recapitulated with the simple administration of IL-12 and IL-18, and more important, that IL-12 and IFN-γ play a critical role in shaping the inflammatory environment for the differentiation of IL-18Rα^+^ ILC into ILC1-like cells.

Lung IL-18Rα^+^ ILCs have been described to differentiate into ILC2 in the context of type 2 inflammation (Ghaedi et al., 2020; Zeis et al., 2020). To further demonstrate that the inflammatory environment shapes the fate of lung IL-18Rα^+^ ILCs, we studied the effect of IL-33, a well-known inducer of both mature and immature ILC2 (Moro et al., 2010; Neill et al., 2010; Price et al., 2010), on ILC1-like differentiation. IL-33 has been shown to enhance ILC2 plasticity (Silver et al., 2016), but its impact on lung IL-18Rα^+^ ILC has not been addressed. IL-33 alone did not induce the differentiation of ILC1-like cells, although it did induce a high expansion of both IL-18Rα^−^ ILC2 and IL-18Rα^+^ ILC (Figures 2H–2J). In association with IL-12 and IL-18, IL-33 was able to enhance ILC1-like differentiation (Figure 2K). Intriguingly, while IL-18Rα^+^ ILC expressed ILC2 markers (ST2, Arg1, and IL-5) in IL-33-treated mice and ILC1-like markers (CD49a, IFN-γ) in IL-12/IL-18-treated animals, the combination of IL-12/IL-18 with IL-33 led to a mixed ILC1/ILC2 phenotype with the capacity to produce both IL-5 and IFN-γ (Figures S3G and 2L). Further characterization of these co-producers showed that IL-5^+^IFN-γ^+^IL-18Rα^+^ ILC displayed a mixed ILC1/ILC2 phenotype (GATA3, T-bet, CD49a) compared to single IL-5^+^ or IFN-γ^+^ producers (Figures S3H–S3J), supporting the notion that these cells undergo differentiation into ILC2 or ILC1-like cells, depending on the inflammatory environment. Because ST2 is expressed by various cell types besides ILC2, we also tested neuromedin U (NMU), whose receptor is solely present in bone marrow ILC2P and in lung ILC2 (Cardoso et al., 2017; Klose et al., 2014; Wallrapp et al., 2017). Like IL-33, NMU potentiated the differentiation of ILC1-like cells induced by IL-12 and IL-18 (Figure S3K). Altogether, these results demonstrate that lung IL-18Rα^+^ ILCs exhibit a highly adaptable polarization potential, depending on the inflammatory environment. While they strengthen the ILC2 response in a type 2 environment, these cells rather differentiate into IFN-γ-producing ILC1-like cells in a type 1 environment. Although we cannot exclude local plasticity of other ILC subsets, or ILCP recruitment from the bone marrow, our results strongly suggest the local differentiation of lung ILC precursors into ILC1-like cells in the context of type 1 inflammation.

### ILC1-like cell differentiation is associated with a metabolic reprogramming toward glycolysis

**Figure 3:**
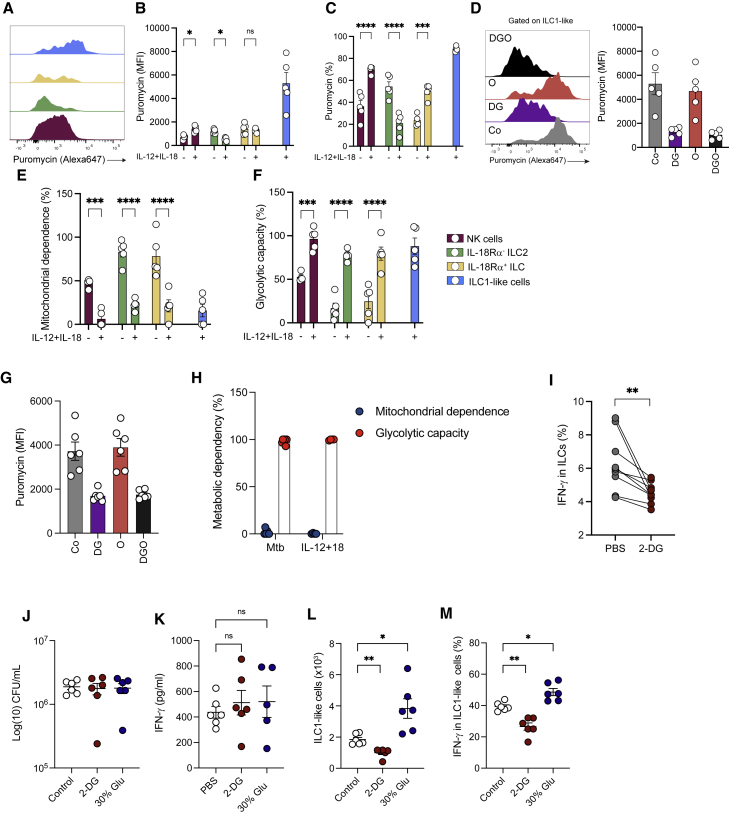
Metabolic reprogramming toward glycolysis is associated with an ILC1-like cell differentiation (A) Representative histograms of puromycin staining in NK cells (violet), IL-18Rα^−^ ILC2 (green), IL-18Rα^+^ ILC (yellow), and ILC1-like cells (blue) in IL-12+IL-18-treated mice. (B) Expression of puromycin (mean fluorescence intensity [MFI]) in NK cells (violet), IL-18Rα^−^ ILC2 (green), IL-18Rα^+^ ILC (yellow), and ILC1-like cells (blue) in PBS versus IL-12+IL-18-treated mice. (C) Percentage of puromycin-positive cells in the indicated ILC subsets in PBS versus IL-12+IL-18-treated mice. (D) Representative histograms of puromycin staining (left) and quantification (MFI, right) in ILC1-like cells from IL-12+IL-18-treated mice after incubation with various metabolic inhibitors (Co, control; DG, 2-deoxyglycose; O, oligomycin; DGO, 2-deoxyglucose + oligomycin). (E and F) Percentages of mitochondrial dependence (E) and glycolytic capacity (F) in the indicated ILC subsets in PBS versus IL-12+IL-18-treated mice. (G) Expression of puromycin (MFI) in ILC1-like cells from *Mtb*-infected Rag2^−/−^ mice after incubation with various metabolic inhibitors. (H) Percentage of mitochondrial dependence (blue) and glycolytic capacity (red) in ILC1-like cells in *Mtb*-infected versus IL-12+IL-18-treated Rag2^−/−^ mice. (I) Expression of IFN-γ in total ILCs from *Mtb*-infected Rag2^−/−^ mice after *ex vivo* stimulation with IL-12+IL-18 in the presence or absence of 2-DG. (J) Mycobacterial loads at day 28 post-infection in 2-DG-treated (red) versus 30% glucose-treated (blue) versus control Rag2^−/−^ mice. (K) Quantification of IFN-γ in the lung supernatant of 2-DG-treated (red) versus 30% glucose-treated (blue) versus control Rag2^−/−^ mice at day 28 post-infection. (L) Absolute numbers of ILC1-like cells in 2-DG-treated (red) versus 30% glucose-treated (blue) versus control Rag2^−/−^ mice at day 28 post-infection. (M) Percentages of IFN-γ ^+^ cells in ILC1-like cells after *ex vivo* stimulation by PMA/ionomycin in 2-DG-treated (red) versus 30% glucose-treated (blue) versus control Rag2^−/−^ mice at day 28 post-infection. Each symbol represents an individual mouse, and statistical analysis was performed using 2-way (B–F) and 1-way (G–M) ANOVA and a paired t test (I). ^∗^, p < 0.05; ^∗∗^, p < 0.01; ^∗∗∗^, p < 0.001; ^∗∗∗∗^, p < 0.0001. Graphs depict data as means ±SEMs from 2 (A–M) independent experiments.

RNA sequencing analyses of intestinal ILCs revealed that each subset displays specific metabolic profiles (Gury-BenAri et al., 2016). While the need for polyamine metabolism for lung ILC2 functions relies on Arg1 (Monticelli et al., 2016), the glycolytic pathway necessary for ILC3 functions depends on the mammalian target of rapamycin (mTOR) and hypoxia-inducible factor 1α (HIF1α) (Di Luccia et al., 2019). However, little is known about the metabolic adaptation of ILCs to their environment during infection (Joseph et al., 2018). Fate decisions of immune cells such as those underlying the differentiation of T regulatory cells (Treg)/Th17 or Treg/Th1 have been tightly associated with metabolic reprogramming (Clever et al., 2016, Dang et al., 2011, Shi et al., 2015). Given that IL-18Rα^+^ ILCs present the ability to differentiate into ILC1-like cells in a type 1 inflammatory context, we investigated the metabolic pathways engaged during ILC1-like cells differentiation. To gain insight in ILC metabolism, we took advantage of the recently described single-cell energetic metabolism by profiling translation inhibition (SCENITH) method (Argüello et al., 2020), which allows us to determine global metabolic dependencies and capacities at the single-cell level. SCENITH uses protein synthesis levels as a readout and is particularly appropriate to analyze the metabolism of rare cells, such as ILCs. ILC1-like cells were compared to control cells known to rely on a glycolytic metabolism (e.g., NK cells) and to ILC2 in lungs. In agreement with the inhibitory effect of type 1 inflammation on IL-18Rα^−^ ILC2 (Figures 1F and S1D), the administration of IL-12 and IL-18 downregulated the ILC2 global level of translation, as assessed via the detection of puromycin incorporation (Figures 3A–3C). Conversely, the level of translation was increased in NK cells, IL-18Rα^+^ ILCs, and ILC1-like cells, with the latter cells displaying the highest rate (Figures 3A–3C). The analysis of protein synthesis in the presence of inhibitors targeting different metabolic pathways, namely 2-deoxyglucose (2-DG) for glycolysis and oligomycin for OXPHOS, allowed us to assess the mitochondrial dependence and glycolytic capacity of the cells (Figure 3D). We found that, in all of the ILC subsets tested, type 1 inflammation led to a global decrease in their mitochondrial dependence, together with an increase in their glycolytic capacity, which is a canonical feature of the Warburg effect (Heiden et al., 2009) (Figures 3E and 3F). Similarly, ILC1-like cells relied on glycolysis rather than mitochondrial OXPHOS for their metabolism when generated after *Mtb* infection (Figures 3G and 3H). Thus, a metabolic reprogramming toward glycolysis is significantly induced in IL-18Rα^−^ ILC2, IL-18Rα^+^ ILCs, and ILC1-like cells upon type 1 inflammation. However, this program was associated with a global inhibition of IL-18Rα^−^ ILC2 compared to the other ILC subsets tested.

Arg1 has been previously identified as a critical component of the metabolic programming of lung ILC2, with its inhibition or genetic inactivation resulting in reduced aerobic glycolysis (Monticelli et al., 2016). In agreement with previous studies (Bando et al., 2013; Monticelli et al., 2016; Schneider et al., 2019), we found that Arg1 was highly expressed in both IL-18Rα^−^ ILC2 and IL-18Rα^+^ ILC (Figures S2A and S2B). However, during type 1 inflammation, the expression of Arg1 decreased in IL-18Rα^+^ ILC to levels similar to those in ILC1-like cells, supporting the idea that Arg1 is not implicated in the metabolic regulation of ILC1-like cell differentiation. In addition, mTOR has been identified as an important metabolic regulator for human ILC2 function in response to IL-33 through the induction of glycolysis (Surace et al., 2021). To determine whether mTOR could play a role in the differentiation of ILC1-like cells, we used tamoxifen-inducible Id2^Cre−ERT2^ mice in combination with a ROSA^tdRFP^ reporter allele (Fiancette et al., 2021), and further crossed these mice with mTOR^fl/fl^ to enable the deletion of mTOR in the ILC compartment after tamoxifen injection. Upon tamoxifen administration, Cre activation was reported via RFP expression among NK1.1^−^ lung ILCs (Figure S4A). Interestingly, no difference was observed in the generation of ILC1-like cells in RFP^+^ cells when comparing Id2^iΔmTOR^ and control Id2^Cre−ERT2^ mice after IL-12+IL-18 treatment, suggesting that the differentiation of ILC1-like cells is mTOR independent (Figures S4B and S4C).

Next, we sought to determine the role of glycolysis in the differentiation of ILC1-like cells during *Mtb* infection. Treatment with 2-DG, an inhibitor of glycolysis, during *ex vivo* stimulation of total lung ILCs decreased the proportion of IFN-γ^+^ ILCs (Figure 3I), showing that IFN-γ production in ILCs is glycolysis dependent. We then assessed the impact of 2-DG treatment *in vivo* and, since glucose is consumed in the lungs of *Mtb*-infected mice (Fernández-García et al., 2020), we also investigated whether glucose supplementation could modulate the differentiation of ILC1-like cells. Although not affecting the bacterial load (Figure 3J) or the amount of IFN-γ detected in the lung supernatant (Figure 3K), 2-DG administration decreased the number of ILC1-like cells as well as their ability to produce IFN-γ (Figures 3L and 3M). In contrast, glucose supplementation in the animals’ drinking water enhanced the differentiation of ILC1-like cells (Figure 3L) and increased the percentage of IFN-γ ^+^ ILC1-like cells (Figure 3M). Thus, these results strongly suggest that glycolysis is required to support ILC1-like cells differentiation and function during *Mtb* infection.

### ILC1-like cells are protective during *Mtb*

**Figure 4:**
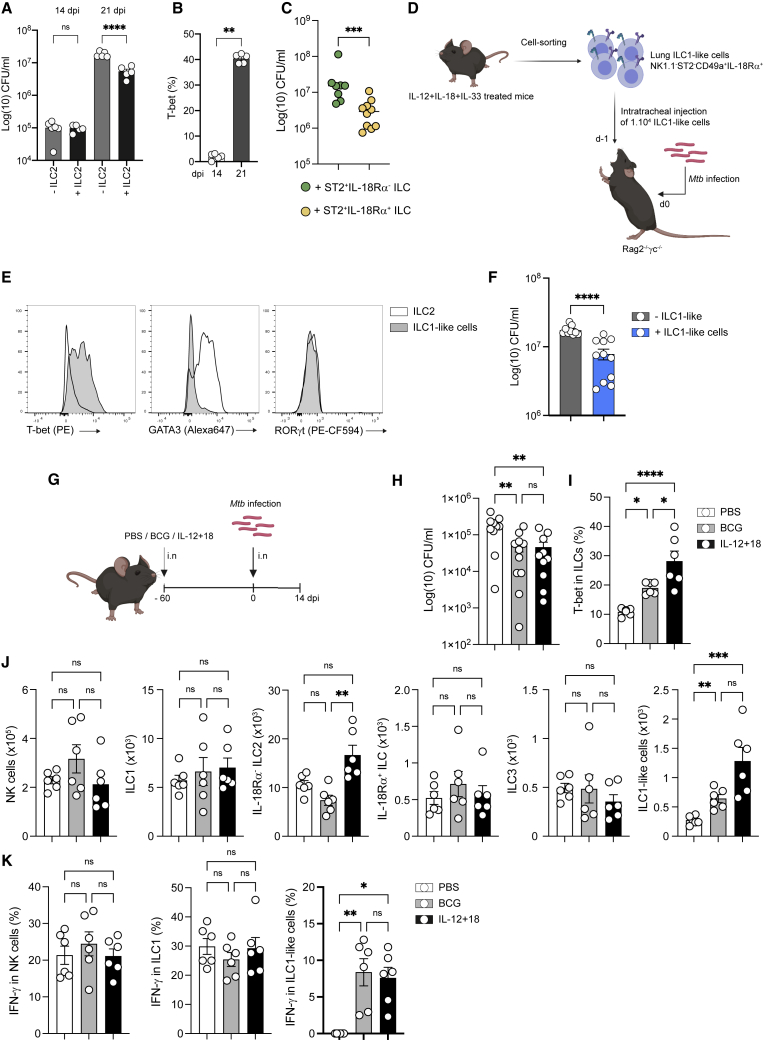
ILC1-like cells confer protection against *Mtb* (A) Mycobacterial loads at days 14 and 21 post-infection in Rag2^−/−^γc^−/−^ mice adoptively transferred with ST2^+^ILC (black) or not (gray). (B) T-bet expression (%) at different days post-infection in transferred ILC2 in Rag2^−/−^γc^−/−^ mice. (C) Mycobacterial loads at day 21 post-infection in Rag2^−/−^γc^−/−^ mice adoptively transferred with ST2^+^IL-18Rα^−^ ILC (green) versus ST2^+^IL-18Rα^+^ ILC. (D) Schematic representation of the *in vivo* expansion of ILC1-like cells in *Rag2*^−/−^ mice treated with IL-12+IL-18+IL-33, cell sorting of ILC1-like cells (Lin^−^CD45.2^+^CD90.2^+^NK1.1^−^ST2^−^CD49a^+^IL-18Rα^+^), and adoptive transfer in *Rag2*^−/−^*γc*^−/−^ 1 day before infection with *Mtb* by the intratracheal route. (E) Representative histograms of T-bet, GATA3, and RORγt expression in sorted ILC1-like cells (gray) versus ILC2 (Lin^−^CD45.2^+^CD90.2^+^NK1.1^−^ST2^+^ cells). (F) Bacterial loads at day 21 post-infection in *Rag2*^−/−^*γc*^−/−^ mice having received (gray) or not (blue) an adoptive transfer of ILC1-like cells from IL-12+IL-18+IL-33-treated *Rag2*^−/−^ mice 1 day before *Mtb* infection. (G) C57BL/6 were vaccinated by intranasal administration of BCG or treated intranasally with IL-12+IL-18 or not (PBS) 60 days before *Mtb* infection. After 14 days post-infection, mice were euthanized. (H) Mycobacterial loads at day 14 post-infection in BCG-vaccinated (gray), IL-12+IL-18-treated (black), or control mice (white). (I) Percentages of total lung ILCs expressing T-bet. (J) Absolute numbers of NK, ILC1, IL-18Rα^−^ ILC2, IL-18Rα^+^ ILC, ILC3, and ILC1-like cells at day 14 post in BCG-vaccinated (gray), IL-12+IL-18-treated (black), or control mice (white). (K) Percentages of IFN-γ ^+^ cells among NK (left), ILC1 (center), and ILC1-like cells (left) in BCG-vaccinated (gray), IL-12+IL-18-treated (black), or control mice (white). after *ex vivo* stimulation with IL-12+IL-18. Each symbol represents an individual mouse. Statistical analysis was performed using the Mann-Whitney test (B–F) and 1-way (H–K) and 2-way (A) ANOVA tests. ^∗^p < 0.05; ^∗∗^p < 0.01; ^∗∗∗^p < 0.001; ^∗∗∗∗^p < 0.0001. Graphs depict data as means ±SEMs. Data are representative of 2 (A, B, and F–K) independent experiments and a pool of 2 experiments (C).

We then addressed the protective role of ILC2 and ILC1-like cells against *Mtb* using an adoptive transfer model. Transfer of purified lung ST2^+^IL-18Rα^+/−^ ILC (Figure S1H) did not lead to a reduction in bacterial loads compared to non-transferred *Rag2*^*−/−*^*γc*^*−/−*^ mice when analyzed at day 14 post-infection. However, a significant reduction in bacterial loads was observed in ST2^+^ ILC-transferred mice at day 21 post-infection (Figure 4A). Interestingly, the increase in T-bet expression among transferred ILCs was observed only after day 21 and not after day 14 (Figure 4B). Furthermore, a more pronounced reduction in bacterial loads was observed in *Rag2*^*−/−*^*γc*^*−/−*^ mice that received IL-18Rα^+^ ILC2 compared to those that received IL-18Rα^−^ ILC2 (Figures 1J and 4C). These results point to the notion that protection was rather due to the differential potential of IL-18Rα^+^ cells into ILC1-like cells rather than to mature ILC2. To directly assess the contribution of ILC1-like cells to protection against *Mtb*, we took advantage of the cytokine-induced ILC1-like cell model (Figure 4D), which included IL-33 to potentiate the response and to generate enough ILC1-like cells for adoptive transfer (see Figure 2K). As expected, these cells expressed T-bet, but not GATA3 or RORγt after sorting (Figure 4E). Remarkably, the transfer of as few as 10,000 ILC1-like cells resulted in a significant reduction in bacterial loads after *Mtb* challenge, demonstrating the protective capacity of ILC1-like cells during *Mtb* infection (Figure 4F).

Since these cells are absent until 21 days after *Mtb* infection (Figure 1G), we investigated whether Bacille Calmette-Guérin (BCG), the only available vaccine for tuberculosis (TB), could induce an early generation of ILC1-like cells in the lung when delivered intranasally, a route providing better protection than the conventional subcutaneous route (Perdomo et al., 2016), before *Mtb* infection (Figure 4G). In addition to the BCG-vaccinated mice, another group of mice was treated with IL-12 and IL-18 intranasally (Figure 4G). As expected, mucosal BCG vaccination induced protection upon *Mtb* challenge as well as the IL-12 and IL-18 treatment (Figure 4H). In both BCG-vaccinated mice and IL-12+IL-18-treated mice, protection correlated with an increase in T-bet expression in ILCs (Figure 4I). More important, although BCG vaccination or IL-12+IL-18 treatment had no impact on other ILC subsets (Figure 4J), higher numbers of ILC1-like cells were detected at 14 days post-infection, a time when IFN-γ -producing ILC1-like cells were virtually absent from non-vaccinated mice (Figure 4K). Overall, BCG vaccination and IL-12+IL-18 treatment both promoted ILC1-like cells in early stages of infection, which could contribute to protection against *Mtb* at an early stage of infection.

## Discussion

How ILCs adapt to environmental cues within tissues is still not fully understood, especially during chronic bacterial infection. Here, we provide evidence that upon *Mtb* infection, an ILC1-like cell population emerges among lung ILCs. While the plastic differentiation of lung ILC2 into ILC1-like cells has previously been reported in the influenza virus infection model, whether ILC1-like cells originate from mature or immature ILC2 was not addressed (Silver et al., 2016). In our model, mature (IL-5 producing) ILC2, as well as NK/ILC1, display a very limited potential to differentiate into ILC1-like cells. As recently described (Ricardo-Gonzalez et al., 2018; Zeis et al., 2020), lung ILC2 is a complex and heterogeneous population of cells with a broad spectrum of maturity. Although the identity of the immature ILC2 population giving rise to ILC1-like cells remains to be formally demonstrated, we found that a lung IL-18Rα^+^ ILC subset, sharing phenotypic similarities with bone marrow ILC2P, retained the ability to differentiate into ILC1-like cells. This population expresses intermediate levels of ILC2 markers (e.g., GATA3, Arg1, ST2), indicating that a lung ILCP skewed toward an ILC2 phenotype. Furthermore, IL-18Rα^+^ ILCs display a hybrid phenotype between ILC1 and ILC2 in the presence of ILC1- (e.g., IL-12, IL-18, IFN-γ) and ILC2- (e.g., IL-33, NMU) activating cytokines. These results sustain the notion that local ILC precursors in the lungs may undergo a context-dependent *ILCpoiesis* (Ghaedi et al., 2020; Zeis et al., 2020). This context may drive the generation of ILC subsets adapted to *Mtb* infection in the lungs. In this regard, type 1 or type 2 environments triggered by cytokines drove the conversion of IL-18Rα^+^ ILC toward mature ILC2 or ILC1-like cells, respectively. We identified IL-12 and STAT1 as critical regulators of ILC1-like cell differentiation. In humans, IL-12 was reported to induce the conversion of blood ILC2 into IFN-γ-producing cells (Lim et al., 2016). Interestingly, IFN-γ is a well-known repressor of ILC2 function through STAT1 signaling (Califano et al., 2018; Duerr et al., 2016; Molofsky et al., 2015; Moro et al., 2016; Stier et al., 2017). Recently, IFN-γ has been identified as a positive, intrinsic regulator of ILC1 differentiation in the liver (Bai et al., 2021). Thus, repression of ILC2 by IFN-γ could promote commitment toward ILC1 differentiation via the reduction in the expression of ILC2-activating signals (e.g., IL-33) or intrinsically through a T-bet program imprinting. A better understanding of the heterogeneity of the lung ILCs at the transcriptional level during type 1 and type 2 immunity will permit the deepening of the identity of these different subsets.

The impact of metabolic reprogramming induced by infections on immune cells is a field of intense investigation, which remains particularly focused on abundant cells, such as T cells and macrophages. In contrast, how less abundant, tissue-resident cells, such as ILCs, adapt to their environment during infection is barely known (Joseph et al., 2018). We report that newly generated ILC1-like cells display a glycolytic metabolism. Such a pathway is also induced in ILC2, but in this case, it is rather associated with their inhibition. Arg1, which is constitutively expressed by lung and bone marrow ILC2 (Bando et al., 2013; Monticelli et al., 2016), was linked to the ability of ILC2 to perform glycolysis and to sustain their functions (Monticelli et al., 2016). However, the induction of HIF1α during ILC2 development in von Hippel-Lindau (VHL)-deficient mice disfavored the acquisition of an ILC2 phenotype (Li et al., 2018). Taken together, these results suggest that cells with a dramatically different fate such as ILC2 and ILC1-like cells may rely on the same global metabolic pathway. In contrast to lung-mature ILC2, Arg1 was not detected in ILC1-like cells, and was reduced in IL-18Rα^+^ ILC upon type 1 inflammation, suggesting an Arg1-independent glycolysis induction. Moreover, while mTOR expression has been associated with glycolysis in NK cells (Marçais et al., 2014) and ILC2 (Salmond et al., 2012; Surace et al., 2021), mTOR depletion in ILC did not prevent ILC1-like differentiation, suggesting the use of different signaling pathways by different cells displaying a glycolytic metabolism. Further investigation to identify the metabolic regulators of these cells would provide insight into the link between metabolism, plasticity, and function of ILCs depending on the inflammatory environment.

Recently, ILC3 were reported to mediate an indirect protection against HN878, a hypervirulent strain of *Mtb* (Ardain et al., 2019). Although our results confirm the expansion and activation of ILC3 during *Mtb* infection (Figures 1F and S1D), we report the expansion of an ILC1-like cell population endowed with protective potential using infection with the laboratory-adapted strain H37Rv. The infection with HN878 is known to induce a different inflammatory pattern (e.g., strong production of IL-1β and type 1 IFN) and protective mechanisms (e.g., IL-17 and IL-22 production) compared to H37Rv (Gopal et al., 2014; Manca et al., 2001). Given that the differentiation of lung IL-18Rα^+^ ILC precursors into ILC1-like is dependent on the inflammatory environment, the impact of different strains of *Mtb* on ILC plasticity remains to be explored further.

In conclusion, we propose that the differentiation of pulmonary IL-18Rα^+^ ILC precursors into ILC1-like cells is regulated by both inflammatory and metabolic environmental cues induced by *Mtb* infection. Our observation that BCG vaccination favors the early generation of ILC1-like cells and that ILC1-like cells are endowed with protective potential during *Mtb* infection paves the way for future studies aimed at elucidating the role played by ILC1-like cells in natural or vaccine-induced protection against TB. More broadly, targeting ILC1-like cells using dedicated strategies may help develop novel approaches to combat TB and other inflammatory diseases.

### Limitations of the study

Although our study provides evidence that the inflammatory environment commits the fate of lung IL-18Rα^+^ ILC to differentiate into protective ILC1-like cells during Mtb infection, several limitations should be mentioned. First, the lack of adequate animal models limited our ability to track the fate of, to selectively deplete lung IL-18Rα^+^ ILC, or to expand specifically IL-18Rα^+^ ILC for adoptive transfer. IL-33 induces the expansion of IL-18Rα^+^ ILC, but likely commits these cells toward an ILC2 lineage, although it retains the potential to differentiate into ILC1-like when placed in a type 1 environment. Such models would be needed to formally demonstrate the role of IL-18Rα^+^ ILC in ILC1-like cell differentiation, as well as to discriminate the intrinsic or extrinsic role of the IL-12/IFN-γ/STAT1 axis. Second, in the absence of a model to selectively deplete ILC1-like cells, we could not formally demonstrate the impact of the early generation of ILC1-like cells after BCG vaccination on the enhanced control of *Mtb*. Finally, the mechanisms used by ILC1-like cells to confer protection remains unknown. Although it is tempting to hypothesize that IFN-γ production plays a role, this hypothesis could not be explored further since IFN-γ is required for the generation of ILC1-like cells. Future development of dedicated mouse models aimed at addressing these questions will foster our understanding of ILC1-like cell biology.

## STAR★Methods

### Key resources table

**Table:** 

REAGENT or RESOURCE	SOURCE	IDENTIFIER
**Antibodies**		

Anti-mouse CD3 (17A2)	BioLegend	Cat#100204; RRID: AB_312661 Cat#100236; RRID: AB_2561456
Anti-mouse CD4 (RM4-5)	BioLegend	Cat#100406; RRID: AB_312691 Cat#100412; RRID: AB_312697
Anti-mouse CD8a (53-6.7)	BioLegend	Cat#100706; RRID: AB_312745
Anti-mouse TCRαβ (H57-597)	BioLegend	Cat# 109206; RRID: AB_313429
Anti-mouse TCRγδ, (GL3)	BioLegend	Cat# 331208; RRID: AB_1575108
Anti-mouse CD11b (M1/70)	BioLegend	Cat# 151503; RRID: AB_2617034
Anti-mouse CD11c (N418)	BioLegend	Cat# 117306; RRID: AB_313775
Anti-mouse F4/80 (BM8)	BioLegend	Cat# 123108; RRID: AB_893502
Anti-mouse Ly6G (1A8)	BioLegend	Cat# 116206; RRID: AB_313775
Anti-mouse TER119 (TER-119)	Biolegend	Cat# 116206; RRID: AB_313707
Anti-mouse FcεRIa (MAR-1)	Thermofisher	Cat# 11-5898-82; RRID: AB_465308
Anti-mouse CD19 (1D3/CD19)	BioLegend	Cat# 152404; RRID: AB_2629813
Anti-mouse B220(RA3-6B2)	BioLegend	Cat# 103206; RRID: AB_312991
Anti-mouse CD49b (DX5)	BioLegend	Cat# 103504; RRID: AB_313027
Anti-mouse CD45.2 (104)	BD Biosciences BioLegend	Cat# 560693; RRID: AB_1727491 Cat# 109831, RRID: AB_10900256)
Anti-mouse CD45.1	BD Biosciences	Cat# 561235; RRID: AB_10611577
Anti-mouse CD90.2 (30-H12)	BioLegend	Cat# 105320; RRID: AB_493725 Cat# 105328,;RRID: AB_10613293
Anti-mouse CD127 (A7R34)	Thermofisher	Cat# 47-1271-82; RRID: AB_1724012
Anti-mouse NK1.1 (PK136)	BioLegend BD Biosciences	Cat# 108706; RRID: AB_313393 Cat# 564143; RRID: AB_2738617
Anti-mouse IL-18Rα (P3TUNYA),	Thermofisher	Cat# 48-5183-82; RRID: AB_1724012
Anti-mouse ST2 (RMST2-2)	BioLegend Thermofisher	Cat# 145313; RRID: AB_2687364 Cat# 145305; RRID: AB_2561916 Cat# 63-9335-80 RRID: AB_2717062)
Anti-mouse CD226 (10E5)	BioLegend	Cat# 133611; RRID: AB_2715975
Anti-mouse CD49a (Ha31/8)	BD Biosciences	Cat# 564863; RRID: AB_2738987
Anti-mouse NKp46 (29A1.4)	BD Biosciences	Cat# 565099; RRID: AB_2739067
Anti-mouse GATA3 (L50-823)	Thermofisher BD Biosciences	Cat# 56-9966-41; RRID: AB_2811898 Cat# 48-9966-41; RRID: AB_2811833 Cat# 560068; RRID: AB_1645316
Anti-mouse T-bet (4B10)	Thermofisher	Cat# 45-5825-80; RRID: AB_953658 Cat# 12-5825-82; RRID: AB_925761 Cat# 25-5825-82; RRID: AB_11042699
Anti-mouse RORγt (Q31-378)	BD Biosciences	Cat# 562684; RRID: AB_2651150
Anti-mouse TCF-1 (S33-966)	BD Biosciences	Cat# 566693; RRID: AB_2869823
Anti-mouse Arg1 (A1exF5)	Thermofisher	Cat# 56-3697-82; RRID: AB_2734833
Anti-mouse Ki-67 (SolA15)	Thermofisher	Cat# 56-5698-80; RRID: AB_2637479
Anti-mouse Eomes (Dan11mag)	Thermofisher	Cat# 50-4875-82; RRID: AB_2574227
Anti-mouse NK1.1 (PK136)	Bio X Cell	Cat# BE0036; RRID: AB_1107737
Anti-mouse IFN-g	Bio X Cell	Cat# BE0055; RRID: AB_1107694
Anti-mouse CD16/32	BioLegend	Cat# 101320; RRID: AB_1574975
Anti-mouse CD45.2	BioLegend	Cat# 109831; RRID: AB_10900256 Cat# 105320; RRID: AB_493725
Anti-puromycin antibodies (Clone R4743L-E8)	CIML, Marseille	RRID: AB_2827926

**Experimental models: Organisms/strains**		

Mouse C56BL/6	Charles River France	C57BL/6N
Mouse Rag2^−/−^ (B6.129-Rag2Tm1Fwa)	Jackson Laboratories	Cat# 008449 RRID:IMSR_JAX:008449
Mouse Rag2^−/−^IL2Rg^−/-^ (C; 129S4-Rag2tm1.1Flv Il2rgtm1.1Flv/J) on	Jackson Laboratories	Cat#:014593 RRID:IMSR_JAX:014593
Mouse STAT1^−/−^ (B6.129S(Cg)-Stat1tm1dlv/J)	Jackson Laboratories	Cat#:012606 RRID:IMSR_JAX:012606
Mouse IL12p40^−/−^ (B6.129S1-*Il12b*^*tm1Jm*^/J)	Jackson Laboratories	#:002693 RRID:IMSR_JAX:002693
Mouse Red5 (B6(C)-Il5tm1.1(icre)Lky/J)n	Jackson Laboratories	Cat.#:030926 RRID:IMSR_JAX:030926
Mouse ROSA26-YFP (B6.129X1-Gt(ROSA)26Sortm1(EYFP)Cos/J)	Jackson Laboratories	Cat#:006148 RRID:IMSR_JAX:006148
Mouse Id2creERT2 (B6.129S(Cg)-*Id2*^*tm1.1(cre/ERT2)Blh*^/ZhuJ)	Jackson Laboratories	Cat#:016222 RRID:IMSR_JAX:016222
Mouse Rosa26-RFP (Gt(ROSA)26Sor^tm1Hjf^)		MGI:3696099
Mouse mTORfl/fl (B6.129S4-*Mtor*^*tm1.2Koz*^/J)	Jackson Laboratories	Cat#:011009 RRID:IMSR_JAX:011009
Mycobacterium tuberculosis/H37Rv	ATCC	Cat# 27294
BCG Danish	ATCC	Cat# 35733

**Software and algorithms**		

FlowJo Software	Treestar	RRID:SCR_008520
BD FACSDiva Software	BD	RRID:SCR_001456
GraphPad Prism V9.2	GraphPad	https://www.graphpad.com

**Chemicals, peptides, and recombinant proteins**		

2 deoxy-glucose (2-DG)	Sigma	Cat# D8375
rmIL-33 (recombinant mouse interleukin-33)	Ozyme	Cat# BLE580504
rmIFNg (recombinant mouse interferon-gamma)	Ozyme	Cat# BLE575306
rmIL-12 (recombinant mouse interleukin-12)	R&D Systems	Cat# 419-ML-050
rmIL-18 (recombinant mouse interleukin-18)	R&D Systems	Cat# 9139-IL-050
rmIL-7 (recombinant mouse interleukin-7)	R&D Systems	Cat# 407-ML-025
rmIL-2 (recombinant mouse interleukin-2)	R&D Systems	Cat# 402-ML-100/CF
Neuromedin U (NMU)	US Biological	Cat# N2171-80E
Live/Dead Fixable Blue	Invitrogen	Cat# L23105
RPMI	Fisher Scientific	Cat# 11544526
Middlebrook 7H9 medium	Difco/Fisher Scientific	Cat# 11753473
Middlebrook 7H11 agar medium	Difco/Fisher Scientific	Cat# 11799042
Albumin-dextrose-catalase, ADC	Difco/Fisher Scientific	Cat# 11718173
Glucose	Sigma-Aldrich	Cat# G7021-5KG
Tamoxifen	Sigma-Aldrich	Cat# T5648
Paraformaldehyde	Fisher Scientific	Cat# 11586711
Collagenase D	Sigma-Aldrich	Cat# 11088882001
DNAse	Sigma-Aldrich	Cat# 10104159001
PMA	Sigma-Aldrich	Cat# P8139-5MG
Ionomycin	Sigma-Aldrich	Cat# I0634-1MG

**Critical commercial assays**		

EasySepTM Mouse ILC2 Enrichment Kit	Stem Cell Technologies	Cat# 19842
Foxp3 transcription factor staining buffer set	ThermoFisher	Cat# 00-5523-00
IFNg ELISA kit	ThermoFisher	Cat# 15511107

### Resource availability

#### Lead contact

Further information and request for resources and reagents should be directed to and will be fulfilled by the lead contact, Dan Corral (dan.corral@nih.gov).

#### Material availability

This study did not generate new unique reagents. There are restrictions to the commercial use of SCENITH due to a pending patent application (PCT/EP2020/060486).

### Experimental model and subject details

#### Mice

Six-to-eight-week-old female C57BL/6 mice were purchased from Charles River Laboratories France (Saint Germain Nuelles, France). *Rag2*^−/−^ (B6.129-Rag2tm1Fwa), STAT1^−/−^ (B6.129S(Cg)-Stat1tm1dlv/J), Red5 mice (B6(C)-Il5tm1.1(icre)Lky/J)n, Rag2^−/−^γ_c_^−/−^ (C; 129S4-Rag2tm1.1Flv Il2rgtm1.1Flv/J) on a C57BL/6 J were bred in our animal facility. ROSA26 ^fl/stopYFP^ mice (B6.129X1-*Gt(ROSA)26Sor*^*tm1(EYFP)Cos*^/J; 006148) were purchased from The Jackson Laboratory through Charles Rivers Laboratory France. IL-12p40^−/−^ mice were obtained through the NIAID-Taconic exchange program. Inducible deletion of mTOR in the ILC lineage was carried out using mice containing *Id2*^creERT2^ (JAX stock #016222) (Rawlins et al., 2009) and *Rosa26*^tdRFP^ (Luche et al., 2007) alleles - as previously described (Fiancette et al., 2021) - in combination with mTOR^fl/fl^ mice (JAX stock #011009) (Risson et al., 2009). All mice were maintained in specific-pathogen-free animal facility at IPBS and all experiments were conducted in strict accordance with French laws and regulations in compliance with the European Community council directive 68/609/EEC guidelines and its implementation in France under procedures approved by the French Ministry of Research and the FRBT (C2EA-01) animal care committee (APAFIS #1269, #3873, #10546, #16529 and #17384).

#### *Mtb* culture, immunization & mouse infections

The laboratory strain of *Mtb*, H37Rv, was grown at 37°C in Middlebrook 7H9 medium (Difco) supplemented with 10% albumin-dextrose-catalase (ADC, Difco) and 0.05% Tyloxapol (Sigma), or on Middlebrook 7H11 agar medium (Difco) supplemented with 10% oleic acid-albumin-dextrose-catalase (OADC, Difco). Six-to eight-week-old mice were anesthetized with a cocktail of ketamine (60 mg/kg, Merial) and xylasine (10 mg/kg, Bayer) and infected intranasally (i.n.) with 1000 CFUs of mycobacteria in 25 μL of PBS containing 0.01% Tween 80. For immunization, C57BL/6 mice were immunized i.n. with 5 × 10^5^ CFU of BCG (Danish) and were challenged 60 days post-vaccination with H37Rv as previously described. All experiments using *Mtb* were performed in appropriate biosafety level 3 (BSL3) laboratory and animal facility.

#### *In vitro* culture of ILC2

Cell sorted ILC2 were incubated in 6-well plates at a density of 300,000 cells per ml for 4 days with IL-2 (25 ng/ml, R&D) and IL-7 (25 ng/ml, R&D) in RPMI (Difco) supplemented with 10% FBS. After 4 days of culture, ILC2 were harvested for adoptive transfer.

### Method details

#### *In vivo* treatments

Mice were injected intraperitoneally (i.p) one day before infection with either 100 μg of mAb to NK1.1 (PK136, BioXcell) or 200 μg of mAb to IFN-γ (BioXcell) or its isotype control and the procedure was repeated twice a week until completion of the experiment and sacrifice of the mice. 2-DG (1g/kg, Sigma) was injected every other day starting from the day of infection and until completion of the experiment. For glucose supplementation, mice were treated with drinking water containing 30% (w/v) glucose (started 1 week before infection until sacrifice). To inducibly delete mTOR in Id2-expressing cells, Id2^CreERT2^ Rosa26^tdRFP^ mice with or without mTOR flox/flox alleles (Id2^iΔmTOR^) received oral gavage of 5mg Tamoxifen in corn oil four times over the course of 10 days. One week later, mice subsequently received four doses of 100ng rIL-12 and rIL-18 intranasally over a 10-day period, following transient anesthesia with inhaled isoflurane, and were culled to harvest tissues one day after the final dose.

#### Adoptive transfer experiments

For the adoptive transfer of total lung ILC2, *in vitro* cultured of ILC2 were harvested after 7 days of culture and 5x10^5^ to 2x10^6^ cells were transferred i.v. in mice anesthetized with isoflurane one day before *Mtb* infection in Rag2^−/−^γ_c_^−/−^. For the adoptive transfer of the IL-18Rα^−^ or IL-18Rα^+^ ILC2 subsets, both subsets were FACS-sorted and cultured *in vitro* for 7 days in complete RPMI supplemented with 10% FBS. At the end of the culture, cells were harvested, and 1 × 10^5^ cells were transferred i.v. in Rag2^−/−^γ_c_^−/−^ mice anesthetized with isoflurane, one day before i.n. *Mtb* infection. For ILC1-like transfer, 1x10^4^ purified ILC1-like were directly transferred via intratracheal (i.t.) route in mice anesthetized with isoflurane one day before *Mtb* infection in Rag2^−/−^γ_c_^−/−^.

#### Lung harvest

Mice were sacrificed any cervical dislocation under isoflurane anesthesia and lungs were harvested aseptically, homogenized using a gentleMACS dissociator (C Tubes, Miltenyi) in HBSS (Difco), and incubated with DNAse I (0.1 mg/mL, Roche) and collagenase D (2 mg/mL, Roche) during 30 min at 37°C under 5% CO2. When indicated, mice received an i.v. injection of labeled anti-CD45 mAb (5μg) 5 min before sacrifice to discriminate between parenchymal and intravascular cells in subsequent flow cytometry analyses. Lung homogenates were filtered on 40 μm cell strainers and centrifuged at 329 × g during 5 min. Supernatants were conserved for cytokine content analysis. Bacterial loads (colony forming units) were determined by plating serial dilutions of the lung homogenates onto 7H10 solid medium (Difco) supplemented with 10% oleic acid-albumin-dextrose-catalase (OADC, Difco). The plates were incubated at 37°C for 3 weeks before bacterial CFUs scoring. In the remaining fraction, red blood cells were lysed in 150 mM NH4Cl, 10 mM KHCO_3_, 0.1 mM EDTA (pH 7.2) for immunological staining.

#### *In situ* expansion of ILC

To expand ILC2, C57BL/6 or *Rag2*^−/−^ mice were treated intranasally (i.n.) with 100 ng of recombinant IL-33 (Biolegend) each day for 5 consecutive days. For the cytokine-based plasticity model, C57BL/6 or *Rag2*^−/−^ mice were treated i.n. with different combinations of cytokines specified in figures legends at day 1, 3, 5, 8 and sacrificed at day 9: 100 ng of IL-12 (R&D), IL-18 (R&D), IL-33 (Biolegend) or 20 μg of NMU (US Biological) per mouse and per instillation.

#### Flow cytometry

To identify mouse ILCs, single-cell suspensions were stained with mAb for known lineages and with mAb discriminating ILC subsets. mAbs for known lineages included CD3 (17A2, Biolegend), CD4 (RM4-5, Biolegend), CD8a (53-6.7, Biolegend), TCRαβ (H57-597, Biolegend), TCRγδ, (GL3, Biolegend) CD11b (M1/70, Biolegend), CD11c (N418, Biolegend), F4/80 (BM8, Biolegend), Ly6G (1A8, Biolegend), TER119 (TER-119, Biolegend), FcεRIa (MAR-1, Biolegend), CD19 (1D3/CD19, Biolegend), B220 (RA3-6B2, Biolegend), and CD49b (DX5, Biolegend). mAbs discriminating ILC subsets included CD45.2 (104, BD), CD90.2 (30-H12, Biolegend), CD127 (A7R34, eBioscience), NK1.1 (PK136, BD Biosciences), IL-18Rα (P3TUNYA, eBioscience), ST2 (RMST2-2, eBioscience), CD226 (10E5, Biolegend), and CD49a (Ha31/8), NKp46 (29A1.4). mAbs for intracellular staining included GATA3 (L50-823, BD Biosciences), T-bet (4B10, eBiosciences), RORγt (Q31-378, BD Biosciences), TCF-1 (S33-966, BD), Arg1 (A1exF5, BD Biosciences), Ki-67 (SolA15, eBiosciences), and Eomes (Dan11mag, eBiosciences). After extracellular staining, cells were fixed and permeabilized (Foxp3 staining kit, eBiosciences) for intracellular staining. Samples from Biosafety Level 3 were inactivated for 2 hours at RT with 4% paraformaldehyde (ThermoFisher Scientific) after extracellular and intracellular staining.

Live/Dead fixable blue (eBiosciences) and mouse FcBlock (BD Biosciences) were used for all flow cytometry experiments. Cell staining was analyzed using LSR Fortessa flow cytometers (BD) and FlowJo software (v10). Cells were first gated in singlets (FSC-H vs. FSC-W and SSC-H vs. SSC-W) and live cells before further analyses.

#### Intracellular cytokines staining

For intracellular cytokines staining of ILCs, single-cell suspensions from lung were incubated at 37°C with Brefeldin A in association or not with PMA (50 ng/ml, Sigma)/Ionomycine (500 ng/ml, Sigma) or 50 ng/ml of IL-12 and IL-18 for 4 hours before being surface stained, fixed and permeabilized (Foxp3 staining kit, eBiosciences). mAbs for cytokines staining included IFN-γ (XMG1.2, Biolegend), IL-17A (TC11-18H10, BD Biosciences), IL-5 (TRFK5, BD Biosciences), and IL-13 (eBio13A, eBiosciences). To block glycolysis during *ex vivo* stimulation, cells were incubated in the presence of 10mM 2-DG (Sigma).

#### ILC enrichment and cell-sorting

Lung ILCs were enriched from lung single-cell suspensions by using the EasySep™ Mouse ILC2 Enrichment Kit (StemCell). After enrichment, cells were stained with lineage mAb (CD3, CD4, CD8α, TCRαβ, TCRγδ, CD19, B220, CD11b, CD11c, F4/80, TER119, FcεRIa, CD49b, Ly6G) and ILC markers (CD90.2, CD45.2, NK1.1, ST2, IL-18Rα, CD49a). ILC2 were purified as Lin^−^CD45.2^+^CD90.2^+^NK1.1^−^ST2^+^IL-18Rα^+/-^ ILC1-like were purified as Lin^−^CD45.2^+^CD90.2^+^NK1.1^−^ST2^−^CD49a^+^IL-18Rα^+^. Cells were sorted using a FACSAria Fusion cytometer (BD, France).

#### SCENITH assay

SCENITH experiments were performed as previously described (Argüello et al., 2020) using the SCENITH kit containing all reagents and anti-puromycin antibodies (requested from www.scenith.com/try-it). Briefly, lung cell suspensions were stimulated for 15 min at 37°C in the presence of the indicated inhibitors of various metabolic pathways then incubated for 30 min with puromycin at 37°C. At the end of the incubation, puromycin was stained with fluorescent anti-puromycin antibodies (Clone R4743L-E8) by flow cytometry and the impact of the various metabolic inhibitors was quantitated as described (Argüello et al., 2020).

#### Quantification of cytokine production by ELISA

Secreted IFN-γ in *Mtb*-infected lung supernatants were measured by ELISA kits, according to the manufacturer’s instructions (BD Biosciences).

### Quantification and statistical analysis

Statistical analyses were performed using GraphPad Prism 9 software. Agostino and Pearson normality tests were performed to determine whether data followed a normal distribution. Unpaired *t* test (for normal data) or Mann-Whitney (for non-normal data) were performed when two samples were compared; ANOVA (for normal data) or Kruskal-Wallis (for non-normal data) tests were performed when more than two samples were compared. For all analyses, ^∗^ indicates p < 0.05, ^∗∗^ indicates p < 0.01, ^∗∗∗^ indicates p < 0.001, and ^∗∗∗∗^ indicates p < 0.0001.

## Data Availability

• All data reported in this paper will be shared by the lead contact upon reasonable request. • This paper does not report original code. • Any additional information required to reanalyze the data reported in this paper is available from the lead contact upon request.

## Supplemental information

Document S1. Figures S1–S4

Document S2. Article plus supplemental information
