# Supplementary material for: ILC precursors differentiate into metabolically distinct ILC1-like cells during Mycobacterium tuberculosis infection
Source: Cell Rep. 2022 Apr 19;39(3):110715. doi: 10.1016/j.celrep.2022.110715 (PMC9043616; doi:10.1016/j.celrep.2022.110715)
Supplement: Document S1. Figures S1–S4 [file mmc1.pdf]

Cell Reports, Volume *39*

Supplemental information

ILC precursors differentiate into metabolically distinct ILC1-like cells during *Mycobacterium tuberculosis* infection

Dan Corral, Alison Charton, Maria Z. Krauss, Eve Blanquart, Florence Levillain, Emma Lefrançais, Tamara Sneperger, Zoï Vahlas, Jean-Philippe Girard, Gérard Eberl, Yannick Poquet, Jean-Charles Guéry, Rafael J. Argüello, Yasmine Belkaid, Katrin D. Mayer-Barber, Matthew R. Hepworth, Olivier Neyrolles, and Denis Hudrisier

Supplementary Figure 1 related to Figure 1. Dynamics of ILCs during Mtb infection in the mouse model

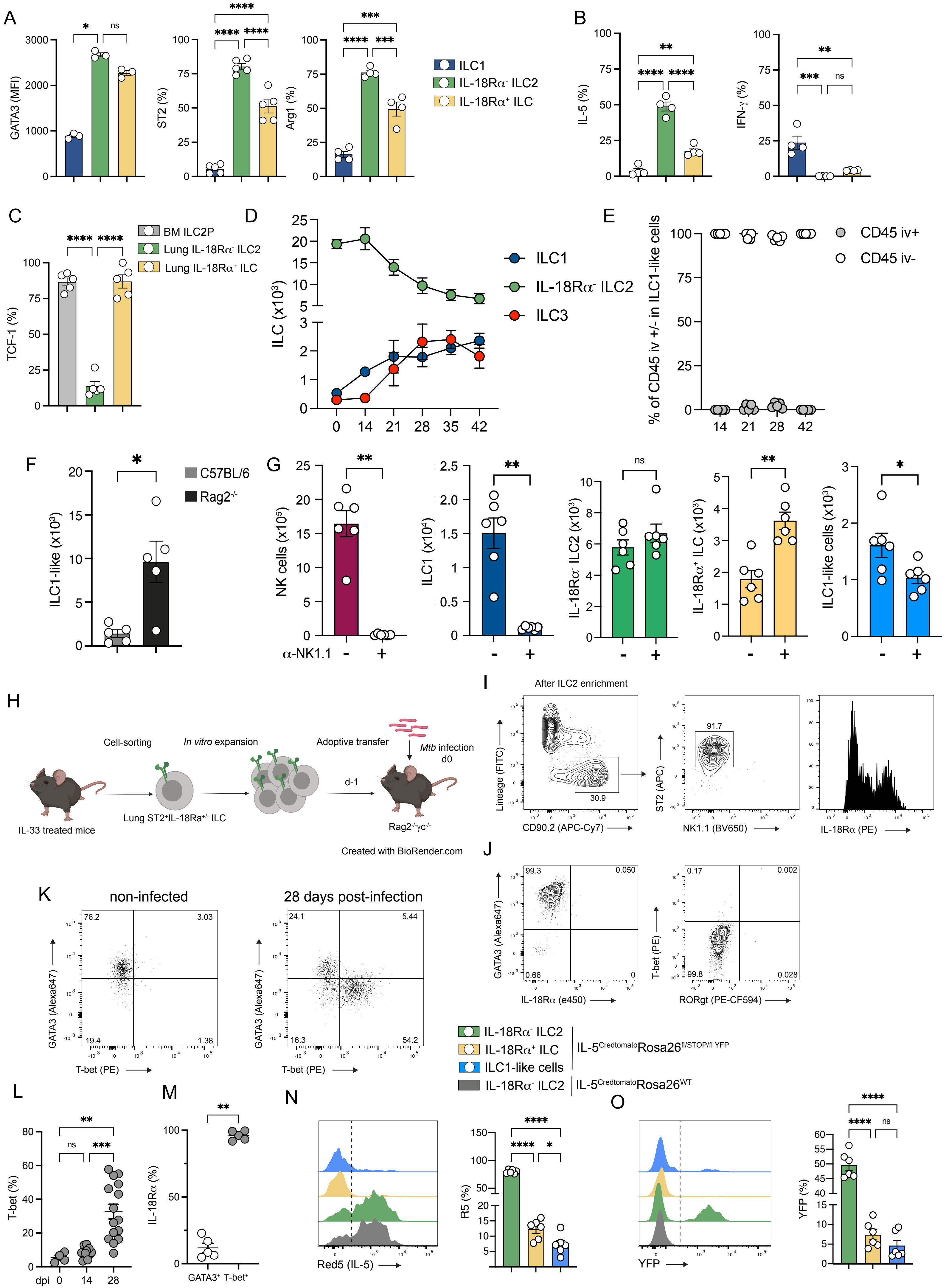

Supplementary Figure 1. Dynamics of ILCs during *Mtb* infection in the mouse model. (A) Expression of GATA3 (MFI), ST2 (%) and Arg1 (%) in ILC1 (blue), IL-18Rα^{-} ILC2 (green) and IL-18Rα^{+} ILC (yellow) at steady-state in the lung of C57BL/6 mice. **(B)** Expression of IL-5 (%) and IFN-γ (%) in the indicated ILC subsets after PMA/ionomycin stimulation at steady-state in the lung of C57BL/6 mice. **(C)** Percentages of TCF-1^{+} cells in ILC2P from bone marrow (grey) compared to IL-18Rα^{-} ILC2 (green) and IL-18Rα^{+} ILC (yellow) from the lungs of C57BL/6 mice at steady-state. **(D)** Absolute numbers of ILC1 (dark blue), ILC3 (red) and IL-18Rα^{-} ILC2 at the indicated days after *Mtb* infection. Prior to sacrifice, mice were injected with fluorescent anti-CD45.2 to distinguish vascular and parenchymal cells. ILC1, ILC3 and IL-18Rα^{-} ILC2 have been gated on lung-resident cells. **(E)** Percentage of CD45.2$^{+/-}$ cells in ILC1-like cells after intravenous injection of fluorescent anti-CD45.2 at the indicated days post-infection. **(F)** Absolute number of ILC1-like cells at day 28 post-infection in C57BL/5 (grey) *vs.* Rag2$^{-/-}$ (black) mice. **(G)** Absolute numbers of NK cells, ILC1, IL-18Rα^{-} ILC2, IL-18Rα^{+} ILC and ILC1-like cells at day 28 post-infection in anti-NK1.1-treated Rag2$^{-/-}$ mice compared to mice treated with isotype control. **(H)** Schematic representation of the *in vivo* expansion of ILC2 in C57BL/6 or *Rag2*$^{-/-}$ mice treated with IL-33, cell-sorting, *in vitro* culture, and adoptive transfer of ILC2 in *Rag2*$^{-/-}$*γc*$^{-/-}$ one day before infection with *Mtb*. **(I)** Gating strategy to purify ILC2 based on the expression of ST2 (left two graphs) and purity of ILC2 after cell-sorting (right). **(J)** Phenotype of ILC2 after *in vitro* culture and before adoptive transfer. **(K)** A representative dot-plot of GATA3 and T-bet expression in Lin^{-}CD45.2^{+}CD90.2^{+} cells isolated from *Rag2*$^{-/-}$*γc*$^{-/-}$ mice adoptively transferred with purified ILC2 then left uninfected (right) or infected with *Mtb* (left). **(L)** Percentages of T-bet expressing ILC at different days post-infection. **(M)** Expression of IL-18Rα (%) in transferred GATA3^{+} (white dots) *vs.* T-bet^{+} (grey dots) ILC at day 28 post-infection in *Rag2*$^{-/-}$*γc*$^{-/-}$mice. (**N-O**) Bar graphs show percentages of IL-5 (**N**) or YFP (**O**) cells in IL-18Rα^{-} ILC2 (green), IL-18Rα^{+} ILC (yellow), and ILC1-like (blue) at day 28 post-infection in IL-5$^{Cre\text{-}dtomato}$ROSA26 $^{fl/stopYFP}$. ILC subsets (Lin^{-}CD45.2^{+}CD90.2^{+} cells) have been defined NK1.1^{-} IL-18Rα^{+}CD49a^{+} for ILC1-like cells, NK1.1^{-}IL-18Rα^{+}CD49a^{-} for IL-18Rα ILC and NK1.1^{-}IL-18Rα^{-}CD49a^{-}ST2^{+} for IL-18Rα^{-} ILC2. Grey histograms represent IL-18Rα^{-} ILC2 from IL-5^{Cre}ROSA26^{WT} from *Mtb*-infected mice as control. Each symbol represents an individual mouse. Statistical analysis was performed using one-way ANOVA test **(A-F, L, N, O)** or Mann-Whitney test **(G, M)** (*, p<0.05; **, P<0.01; ***, p<0.001; ****, p<0.0001). Graphs depict data as mean (± s.e.m). Data are representative of five **(D, E)**, three **(A, B, F, K-M)** and two **(C, G, N, O)** independent experiments.

Supplementary Figure 3 related to Figure 2. The inflammatory environment shapes the fate of IL-18Rα⁺ ILC

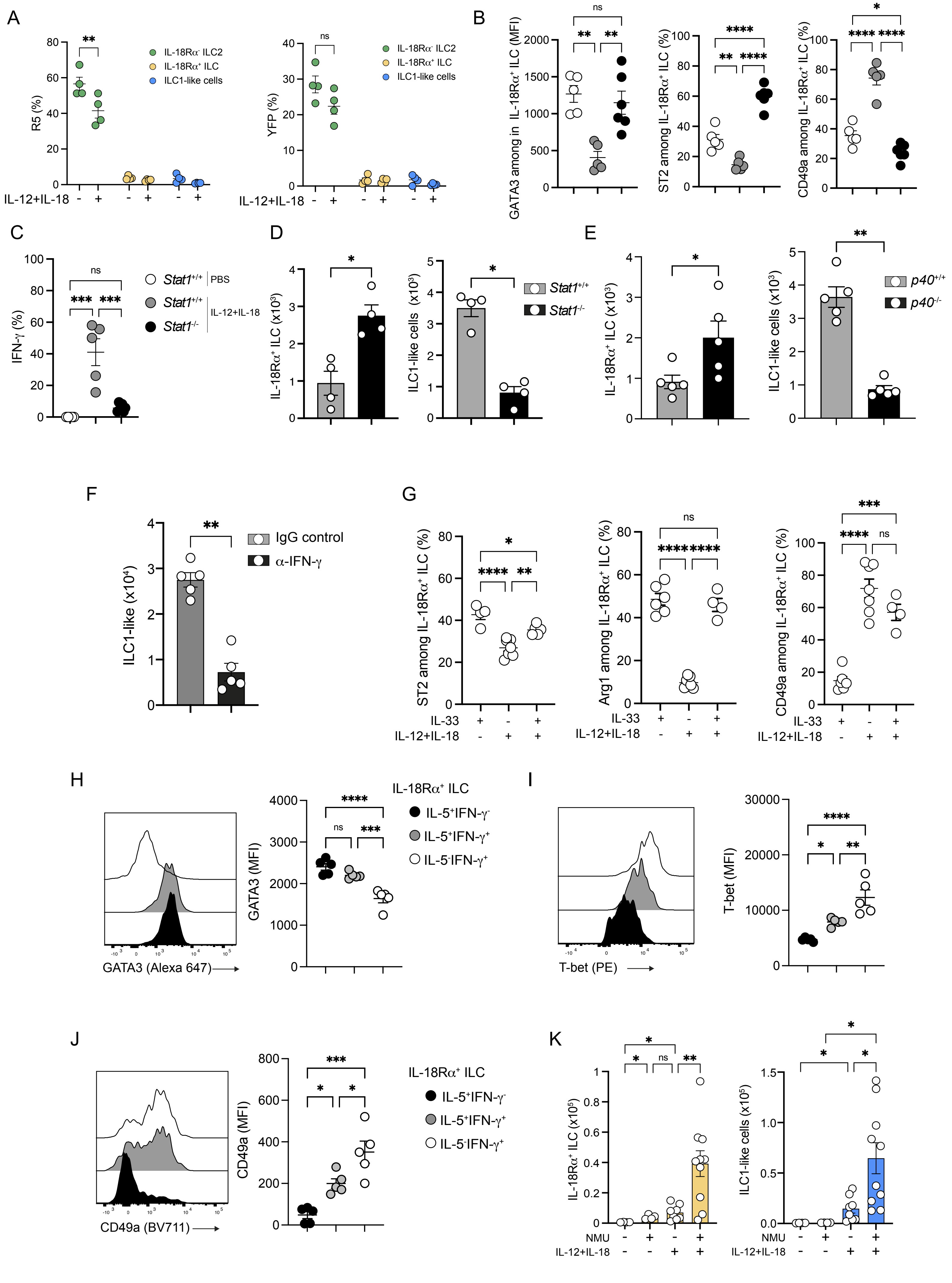

Supplementary Figure 2. IL-12 and IL-18 treatment mimics *Mtb* infection for ILC1-like cell differentiation. (A) Unsupervised t-SNE representation of the expression of different markers (GATA3, Arg1, T-bet, IL-18Rα, CD49a, CD226 and Ki67) on $Lin^{-}CD45.2^{+}CD90.2^{+}NK1.1^{-}ROR\gamma t^{-}$ in non-infected *vs*. *Mtb*-infected (28 dpi) *vs.* IL-12+IL-18 treated $Rag2^{-/-}$ mice. (**B**) Expression (from top to the bottom) of GATA3 (MFI), ST2 (%), Arg1 (%), CD49a (%), CD226 (%) and Ki67 (%) of $IL\text{-}18R\alpha^{-}$ ILC2, $IL\text{-}18R\alpha^{+}$ ILC, and ILC1-like cells in *Mtb*-infected (grey) *vs.* IL-12+IL-18-treated (black) *vs.* Control (white) $Rag2^{-/-}$ mice. Each symbol represents an individual mouse and statistical analysis was performed using two-way ANOVA **(B)** (*, p<0.05; **, P<0.01; ***, p<0.001; ****, p<0.0001). Graphs depict data as mean (± s.e.m) from two **(B)** independent experiments.

Supplementary Figure 2 related to Figure 2. IL-12 and IL-18 treatment mimics Mtb infection for ILC1-like differentiation

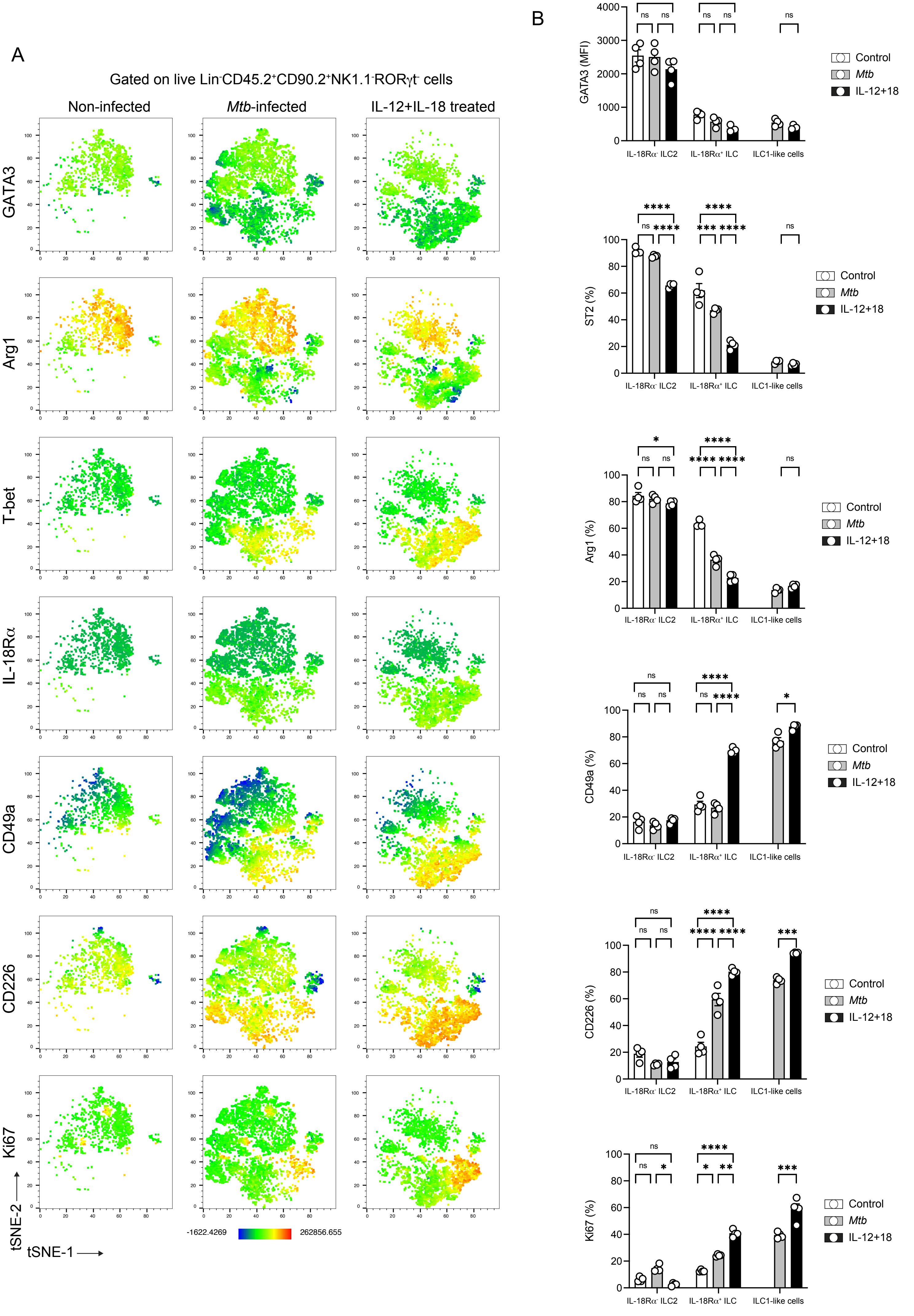

Supplementary Figure 3. The inflammatory environment shapes the fate of IL-18Rα$^+$ ILC. (A) Percentages of cells expressing IL-5 (left) and YFP (right) among IL-18Rα$^-$ ILC2 (green), IL-18Rα$^+$ ILC (yellow), and ILC1-like (blue) cells from IL-5$^{Cre\text{-}dtomato}$ROSA26 $^{fl/stopYFP}$ mice treated with PBS *vs*. IL-12+IL-18. **(B)** Expression of GATA3 (MFI), ST2 (%) and CD49a (%) in IL-18Rα$^+$ ILC in PBS (white dots) *vs*. IL-12+IL-18 treated STAT1$^{+/+}$ (grey dots) and STAT1$^{-/-}$ (black dots) mice. (**C**) as in (**B**) but for the percentages of IFN-γ in IL-18Rα$^+$ ILC after PMA/ionomycin stimulation **(D)** Absolute numbers of IL-18Rα$^+$ ILC (left) and ILC1-like cells (right) in *Mtb*-infected STAT1$^{+/+}$ (grey) and STAT1$^{-/-}$ (black) mice at day 28 post-infection. (**E**) Absolute numbers of IL-18Rα$^+$ ILC (left) and ILC1-like cells (right) in *Mtb*-infected IL-12p40$^{+/+}$ (grey) and IL-12p40$^{-/-}$ (black) mice at day 28 post-infection. (**F**) Absolute numbers of ILC1-like cells in Rag2$^{-/-}$ mice treated with IL12+IL-18 in the presence of a blocking anti-IFN-γ mAb or its isotype control. **(G)** Expression of ST2 (%), Arg1 (%) and CD49a (%) in IL-18Rα$^+$ ILC in Rag2$^{-/-}$ mice treated with IL-33 (black dots), IL-12+IL-18 (white dots) or IL-12+IL-18+IL-33 (grey dots). **(H-J)** Histogram (left) and quantification (right) of GATA3 (G), T-bet (H) and CD49a (I) expression (MFI) in IL-5$^+$IFN-γ$^-$ (black), IL-5$^+$IFN-γ$^+$ (grey), and IL-5$^-$IFN-γ$^+$ (white) IL-18Rα$^+$ ILC obtained from IL-12+IL-18+IL-33-treated Rag2$^{-/-}$ mice. **(K)** Absolute numbers of IL-18Rα$^+$ ILC (left) and ILC1-like cells (right) after intranasal administration of PBS (control), neuromedin U (NMU), IL-12+IL-18, or IL-12+IL-18 +NMU. Statistical analysis was performed using Mann-whitney (**D, E, F**), two-way (**A**), and one-way (**B, C, G-K**) ANOVA test (*, $p<0.05$; **, $P<0.01$; ***, $p<0.001$; ****, $p<0.0001$). Graphs depict data as mean (± s.e.m). Data are representative of two **(A-D, F-K)** independent experiments and one experiment (**E**).

Supplementary Figure 4 related to Figure 3. ILC1-like cell differentiation is mTOR-independent

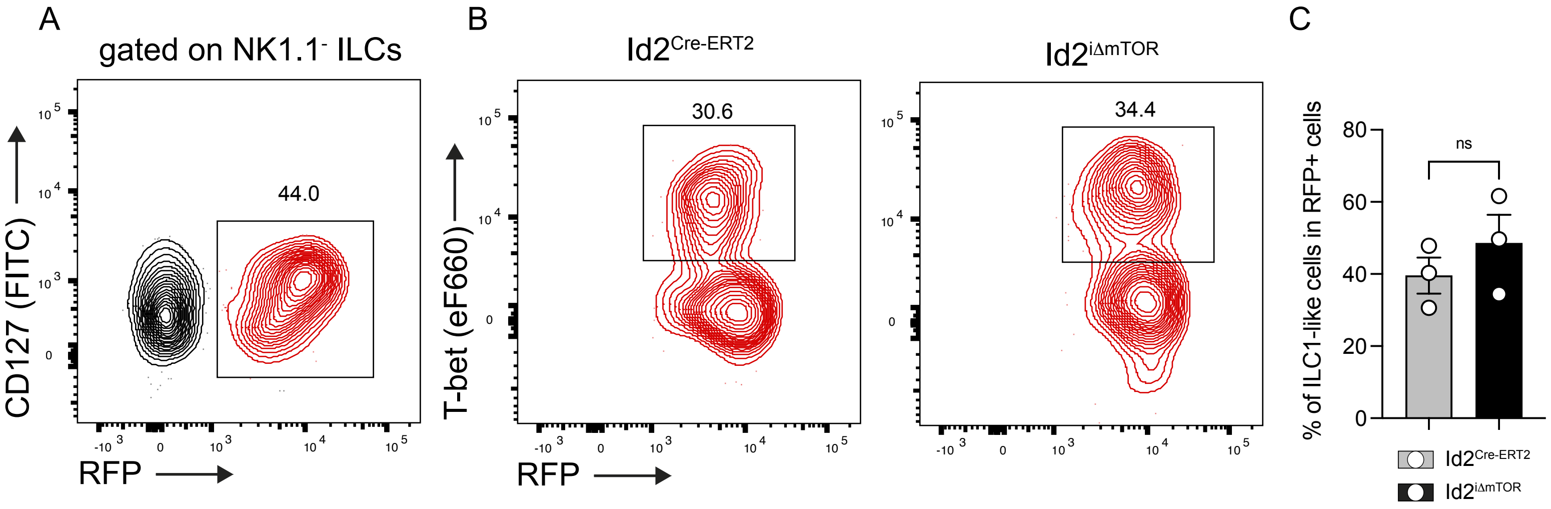

Supplementary Figure 4. ILC1-like cell differentiation is mTOR-independent. (**A**) Representative dot plot of RFP$^+$ in NK1.1$^-$ILCs (Lin$^-$CD45.2$^+$CD90.2$^+$ cells) in IL-12+IL-18-treated Id2$^{Cre-ERT2}$Rosa26$^{fl/RFP}$ following tamoxifen administration. **(B)** Expression of T-bet by RFP$^+$NK1.1$^-$ILCs in IL-12+IL-18-treated control mice (Id2$^{Cre-ERT2}$) and mice further containing a floxed mTOR allele (Id2^{imTOR}) following tamoxifen injection. **(C)** Percentage of ILC1-like cells in RFP$^+$NK1.1$^-$ILCs in IL-12+IL-18-treated Id2$^{Cre-ERT2}$ vs. Id2^{imTOR} mice. Each symbol represents an individual mouse and statistical analysis was performed using Mann-Whitney test. Graphs depict data as mean (± s.e.m) from two independent experiments.
